# Association of Dietary Patterns with MRI Markers of Hepatic Inflammation and Fibrosis in the MAST4HEALTH Study

**DOI:** 10.3390/ijerph19020971

**Published:** 2022-01-16

**Authors:** Athina I. Amanatidou, Andriana C. Kaliora, Charalampia Amerikanou, Stefan Stojanoski, Natasa Milosevic, Chara Vezou, Mirjana Beribaka, Rajarshi Banerjee, Ioanna-Panagiota Kalafati, Ilias Smyrnioudis, Mary Jo Kurth, Aimo Kannt, M. Pilar Francino, Sophie Visvikis-Siest, Panos Deloukas, Carlos Llorens, Fernando Marascio, Natasa Milic, Milica Medic-Stojanoska, Amalia Gastaldelli, Maria Giovanna Trivella, George V. Dedoussis

**Affiliations:** 1 Department of Nutrition and Dietetics, School of Health Science and Education, Harokopio University, 17671 Athens, Greece; amerikanou@windowslive.com (C.A.); vezouch@gmail.com (C.V.); nkalafati@gmail.com (I.-P.K.); dedousi@hua.gr (G.V.D.); 2 Center for Diagnostic Imaging, Oncology Institute of Vojvodine, 21204 Sremska Kamenica, Serbia; stefan.stojanoski@mf.uns.ac.rs; 3 Faculty of Medicine, University of Novi Sad, 21000 Novi Sad, Serbia; natasa.milosevic@mf.uns.ac.rs (N.M.); milnat@libero.it (N.M.); milica.medic1@gmail.com (M.M.-S.); 4 Department of Biology, Faculty of Technology Zvornik, University of East Sarajevo, 75400 Zvornik, Bosnia and Herzegovina; mirjana.beribaka@tfzv.ues.rs.ba; 5 Perspectum Ltd., Oxford OX4 2LL, UK; rajarshi.banerjee@perspectum.com; 6 Chios Mastic Gum Growers Association, 82100 Chios, Greece; ismyrnioudis@gummastic.gr; 7 Clinical Studies Group, Randox Laboratories Ltd., Crumlin BT29 4RN, UK; maryjo.kurth@Randox.com; 8 Fraunhofer Institute for Translational Medicine and Pharmacology ITMP, 60596 Frankfurt, Germany; aimo.kannt@itmp.fraunhofer.de; 9 Area de Genòmica i Salut, Fundació per al Foment de la Investigació Sanitá ria I Biomèdica de la Comunitat Valenciana (FISABIO-Salut Pú blica), 46020 Valencia, Spain; mpfrancino@gmail.com; 10 CIBER en Epidemiología y Salud Pú blica, 28029 Madrid, Spain; 11 INSERM UMR U1122, IGE-PCV, Faculté de Pharmacie, Université de Lorraine, 30 Rue Lionnois, 54000 Nancy, France; sophie.visvikis-siest@inserm.fr; 12 William Harvey Research Institute, Barts and The London School of Medicine and Dentistry, Queen Mary University of London, London EC1M 6BQ, UK; p.deloukas@qmul.ac.uk; 13 Centre for Genomic Health, Life Sciences, Queen Mary University of London, London E1 2AD, UK; 14 Biotechvana, Parc Científic, Universitat de València, Paterna, 46010 Valencia, Spain; carlos.llorens@biotechvana.com; 15 Intervideo Web Service, 88100 Catanzaro, Italy; newvideo@newvideo.net; 16 Clinic for Endocrinology, Diabetes and Metabolic Diseases, Clinical Centre of Vojvodina, 21000 Novi Sad, Serbia; 17 Institute of Clinical Physiology National Research Council, 56124 Pisa, Italy; amalia@ifc.cnr.it (A.G.); trivella@ifc.cnr.it (M.G.T.); 18 ASST Grande Ospedale Metropolitano Niguarda, 20162 Milan, Italy

**Keywords:** NAFLD, NASH, MRI, dietary patterns, MAST4HEALTH

## Abstract

Whereas the etiology of non-alcoholic fatty liver disease (NAFLD) is complex, the role of nutrition as a causing and preventive factor is not fully explored. The aim of this study is to associate dietary patterns with magnetic resonance imaging (MRI) parameters in a European population (Greece, Italy, and Serbia) affected by NAFLD. For the first time, iron-corrected T1 (cT1), proton density fat fraction (PDFF), and the liver inflammation fibrosis score (LIF) were examined in relation to diet. A total of 97 obese patients with NAFLD from the MAST4HEALTH study were included in the analysis. A validated semi-quantitative food frequency questionnaire (FFQ) was used to assess the quality of diet and food combinations. Other variables investigated include anthropometric measurements, total type 2 diabetes risk, physical activity level (PAL), and smoking status. Principal component analysis (PCA) was performed to identify dietary patterns. Six dietary patterns were identified, namely “High-Sugar”, “Prudent”, “Western”, “High-Fat and Salt”, “Plant-Based”, and “Low-Fat Dairy and Poultry”. The “Western” pattern was positively associated with cT1 in the unadjusted model (beta: 0.020, *p*-value: 0.025) and even after adjusting for age, sex, body mass index (BMI), PAL, smoking, the center of the study, and the other five dietary patterns (beta: 0.024, *p*-value: 0.020). On the contrary, compared with low-intake patients, those with medium intake of the “Low-Fat Dairy and Poultry” pattern were associated with lower values of cT1, PDFF, and LIF. However, patients with a “Low-Fat Dairy and Poultry” dietary pattern were negatively associated with MRI parameters (cT1: beta: −0.052, *p*-value: 0.046, PDFF: beta: −0.448, *p*-value: 0.030, LIF: beta: −0.408, *p*-value: 0.025). Our findings indicate several associations between MRI parameters and dietary patterns in NAFLD patients, highlighting the importance of diet in NAFLD.

## 1. Introduction

Non-alcoholic fatty liver disease (NAFLD) is the most common chronic liver disease worldwide. It is an umbrella term for a variety of pathological conditions ranging from simple hepatic steatosis (SS) or non-alcoholic fatty liver (NAFL) to the more severe nonalcoholic steatohepatitis (NASH) and NASH cirrhosis [1]. Research advances in the last decade have demonstrated that NAFLD is a multisystem disease with many complex processes involved in its manifestation and development. Furthermore, an increasing number of studies demonstrate that NAFLD affects a variety of extrahepatic organs and regulatory pathways [2].

NAFLD is a severe public health issue in both industrialized and developing countries, with an estimated global incidence of 25% [3]. The prevalence of NASH varies from 1.5% to 6.45%, with 41% of those with NASH progressing to fibrosis [3]. NASH-related cirrhosis has become the second largest cause of liver transplantation (LT) in the United States since 2013, and it is anticipated to overtake LT as the primary cause in Europe within the next decade [4,5].

NAFLD is closely related to metabolic disorders (such as hypertension, type 2 diabetes) and insulin resistance (IR); in addition, obesity and increased central adiposity are strong indicators of its presence [6,7]. Growing evidence suggests that NAFLD and the related metabolic disorders are linked to an increased risk of cardiovascular disease (CVD) morbidity and mortality [8]. Although there are no definite approved pharmacotherapies for NAFLD, and even though bariatric surgery in morbidly obese NAFLD patients is offered, lifestyle interventions remain the safest and most effective treatment approaches for NAFLD [9,10,11,12,13,14,15]. The high prevalence of the disease has been associated with poor dietary habits [16], and as such, dietary modifications consist of key factors for NAFLD management [17,18,19]. Diets that are high in saturated fats and refined carbohydrates, particularly fructose, and along with physical inactivity, are characterized as predictive mediators for NAFLD [20,21,22]. In contrast, the Mediterranean dietary pattern, which is rich in nuts, whole grains, fruits, vegetables, fish, and olive oil, has been characterized as the diet of choice to ameliorate NAFLD [23].

Numerous studies have described the link between foods and nutrients and the risk of NAFLD [24,25,26,27,28]. In addition, several studies have explored the association of dietary patterns and NAFLD risk. In a recent review and meta-analysis that included 18 studies, the Western dietary pattern was associated with a higher risk, whereas the Prudent and Mediterranean dietary pattern were associated with a lower risk of NAFLD, respectively [29]. Our study aims to assess for the first time the relation of dietary patterns in a NAFLD (MAST4HEALTH) population with novel parameters that quantify hepatic inflammation and fibrosis.

## 2. Materials and Methods

### 2.1. Study Design and Patients

Data included in this study were derived from the MAST4HEALTH project, as described previously [30]. Briefly, 98 participants were recruited across three centers (the Department of Dietetics and Nutritional Science, Harokopio University, Athens, Greece (HUA), Consiglio Nazionale delle Ricerche Institute of Clinical Physiology, Milano section at Niguarda Hospital Italy, (CNR) and Faculty of Medicine, University of Novi Sad, Serbia (UNS)), based on the previously reported inclusion and exclusion criteria [30]: The participants were men and women between the ages of 18 and 67, with a body mass index (BMI) equal or greater than 30 kg/m^2^ and had established NAFLD/NASH as determined by the sensitive LiverMultiScan magnetic resonance imaging (MRI) method (Perspectum Ltd., Oxford, UK). All centers obtained approvals by their ethical committees [30], and the trial was carried out in accordance with the norms of the Declaration of Helsinki and the Data Protection Act of 1998. To take part in the study, all participants provided written informed permission. ClinicalTrials.gov provides access to the trial’s entire protocol (Clinicaltrials.gov MAST4HEALTH, Identifier: NCT03135873).

### 2.2. Measures

Dietary intake was evaluated using a 24-h recall record (3 random days) and data were processed utilizing the Nutritionist Pro™ software version 7.1.0 (Axxya Systems LLC, Stafford, TX, USA) for the estimation of caloric intake. A standardized semi-quantitative food frequency questionnaire (FFQ) was applied to evaluate dietary patterns. This FFQ included 69 questions detailing the frequency of consumption of main food groups and beverages [31]. The frequency was measured using a 6-grade scale ranging from “never/rarely”, “1–3 times/month, 1–2 times/week, 3–6 times/week, 1 times/day,” to “≥2 times/day”, which were subsequently transformed as servings per week. Sixty-six food items were aggregated into 25 food groups (Table S1), [32,33].

Other variables used in the present study included MRI parameters, anthropometric measurements, total type 2 diabetes risk, physical activity, and smoking status. MRI parameters included iron-corrected T1 (cT1), proton density fat fraction (PDFF), and the liver inflammation fibrosis score (LIF). 

Body weight was calculated to the closest 0.1 kg. Height was estimated to the closest millimeter and BMI was calculated as weight (kg) divided by height (m)^2^. In addition, liver enzymes (γ-glutamyltransferase (g-GT), aspartate transaminase (AST), and alanine transaminase (ALT)), lipids (total cholesterol, high-density lipoprotein (HDL), low-density lipoprotein (LDL), and triglycerides (TG)), glucose, insulin, homeostasis model assessment (HOMA-IR), and 75-g of the glucose 2 h oral glucose tolerance test (OGTT) were measured [30]. HOMA-IR was measured using the following formula: fasting glucose (mg/dL) × (fasting insulin)/405. Biochemical measurements (liver enzymes, lipid profile, glucose, insulin, and OGTT) were performed in the pathology labs of the hospitals where the clinical trial took place during the screening of the patients. The established Finnish diabetic risk score (FINDRISK) questionnaire, which includes questions of age, BMI, waist circumference (WC), physical activity, vegetables and fruits consumption, hypertension, and personal and family history of hyperglycemia, was used for the estimation of the total type 2 diabetes risk [34]. The international physical activity questionnaire (IPAQ) [35] was used to assess physical activity level (PAL) and the metabolic equivalent task minutes per week (MET-min/week) were obtained using the IPAQ scoring system. The total physical activity score was calculated by adding all METs. Participants were questioned concerning their smoking behaviors and classified as smokers and non-smokers.

### 2.3. Statistical Analysis

The data handling and analyses were performed using R version 3.5.1 programming (R Foundation, Vienna, Austria) language. The eligibility criteria (Kaiser-Meyer Olkin (KMO) index and Bartlett’s test for sphericity) were examined to test the sample adequacy before applying a principal component analysis (PCA) for the extraction of dietary patterns of the study population. The input data were a correlation matrix of 25 food groups. KMO was 0.71 and the Bartlett’s test was significant at *p* < 0.001, implying the dataset’s suitability for PCA. Using the scree plot method and Kaiser’s criterion (Eigenvalue 1.00), a 6-component solution was chosen. To obtain optimal non-correlated components (dietary patterns), the orthogonal rotation (varimax option) was applied. Food groups with a loading coefficient of ≥|0.35| were identified for each factor as a measure of the significant relevance of a given variable to a given factor. The dietary patterns’ scores identify individual participant adhesion to the pattern. Dietary patterns were labeled with food groups that were positively loaded on the factor. The dietary pattern’s scores were further classified into tertiles, with the first tertile indicating low intake and the third indicating high adherence to the dietary pattern.

The baseline study characteristics and nutrient intake were summarized based on tertiles of dietary patterns. The Shapiro–Wilk test was used to assess the distribution of the variables, which are presented as mean ± standard deviation (SD) for all normally distributed variables (parametric variables) (Shapiro–Wilk *p*-value > 0.05) or as the median and interquartile range (IQR) for all variables that did not follow the normal distribution (non-parametric variables). The analysis of variance (ANOVA) applying Tukey’s post hoc test in the case of parametric variables and the Kruskal–Wallis test applying Dunn’s post hoc test in case of non-parametric variables, was used to assess differences between the dietary patterns’ tertiles. The chi-square test was used to compare tertiles of categorical variables represented as numbers.

Linear regression models were used to evaluate the association between the dietary patterns’ tertiles and the MRI parameters (cT1, PDFF and LIF). The low tertile of each dietary pattern was used as a reference group. Due to the skewness of the distribution, the cT1 and PDFF were log transformed. Four adjustment sets were considered: Model 1—crude; Model 2—adjusted for age + sex; Model 3—adjusted for age + sex + BMI; and Model 4—adjusted for age + sex + BMI + PAL + smoking + center of the study + the other five dietary patterns; Model 5—adjusted for age + sex + BMI + PAL + smoking + center of the study + alcohol intake [yes/no] + the other five dietary patterns. Moreover, the aforementioned regression models were used to assess the association between the dietary pattern’s scores and the MRI-derived biomarkers. A *p*-value < 0.05 was considered significant in all tests.

## 3. Results

Ninety-seven NAFLD patients for whom dietary data were available were included in the current analysis, 68 of whom are males and 29 are females. The patients recruited at three centers (Greece: GR, Italy: IT and Serbia: SR) were compared based on demographic, anthropometric, lifestyle, MRI, and biochemical characteristics (Table 1). BMI was found significantly higher in Greek patients compared to Italians and Serbians (*p*-value: 0.003). In addition, PAL was significantly higher in Serbian patients compared to Italians and Greeks (*p*-value: 0.007). Greek patients had significantly higher LIF in comparison with Italians (*p*-value: 0.019). Italian patients exhibited significantly higher levels of AST (*p*-value: 0.012) and ALT (*p*-value: 0.004) compared with Greeks. A total of 120 min OGTT glucose was significantly higher in Serbian patients than in Greeks and Italians (*p*-value: 0.007). Italian patients had higher levels of HOMA-IR (*p*-value: 0.018) and insulin levels (*p*-value: 0.049) than Serbians.

Six different dietary patterns (components) accounted for 56% of the samples’ total variance identified by PCA (Table 2). Higher absolute factor loadings implied greater contribution to each component. As a result, the following dietary patterns were identified: “High-Sugar” explained 14% of the variance, and the foods identified included pies, dried fruits, processed meat, fruit juice, sweets, and fruits; “Prudent” explained 13% of the variance and included sea-food, fish, eggs, vegetable fat, vegetables, coffee, and tea; “Western” explained 8% of the variance and included refined grains, red meat, and fast food; “High-Fat and Salt” explained 8% of the variance and included salty snacks, sauces, dairy (high-fat), animal and hydrogenated fats, and soft drinks; “Plant-Based” explained 7% of the variance and included whole grains, pulses, and nuts; “Low-Fat Dairy and Poultry” explained 6% of the variance and included dairy (low-fat) and poultry.

This table enables the factor loadings for the 25 food groups that were derived from the PCA analysis. These values describe how much each food group contributes to a particular dietary pattern. The largest the loading the highest the relationship of each food group to the specific pattern. An often-used threshold (|0.35|) was utilized for identifying factor loadings as key contributors to a pattern and labeling these patterns according to the food groups with the highest factor loadings.

The comparisons of demographic, anthropometric, lifestyle, MRI, and biochemical parameters for the different levels of dietary patterns are described in Table S2. In the “Western” pattern, the group with the medium scores had significantly lower ALT levels in comparison to the other two groups (*p*-value: 0.02449). Moreover, in the “Plant-Based” pattern, the AST (*p*-value: 0.04727) and ALT (*p*-value: 0.0138) levels of the medium adherence scores were also lower compared to the other two groups. In addition, the AST/ALT ratio (*p*-value: 0.04029) was significantly lower in the “Plant-Based” pattern of low scores compared to the group of medium scores. Italian patients were found to have higher adherence to the “Prudent” pattern. In addition, Greek patients were found to have more adherences to groups with the highest scores of the “Western” and “Plant-Based” pattern. Moreover, PDFF (%) was detected with significantly higher value in the group with low scores of the “Low-Fat Dairy and Poultry” pattern compared to the medium group (*p*-value: 0.04452).

The differences in nutrient intake between tertiles of dietary patterns are described in Table S3. For example, total sugar (g) (*p*-value: 0.01003) and glucose (g) (*p*-value: 0.008712) intakes were significantly higher in the medium tertile of the “High-Sugar” pattern when compared to intakes in the low tertile but not when compared to the high. When compared to the low tertile, fructose (g) intake was significantly higher in the medium and high tertiles of “High-Sugar” pattern (*p*-value: 0.005621). 

**Figure 1:**
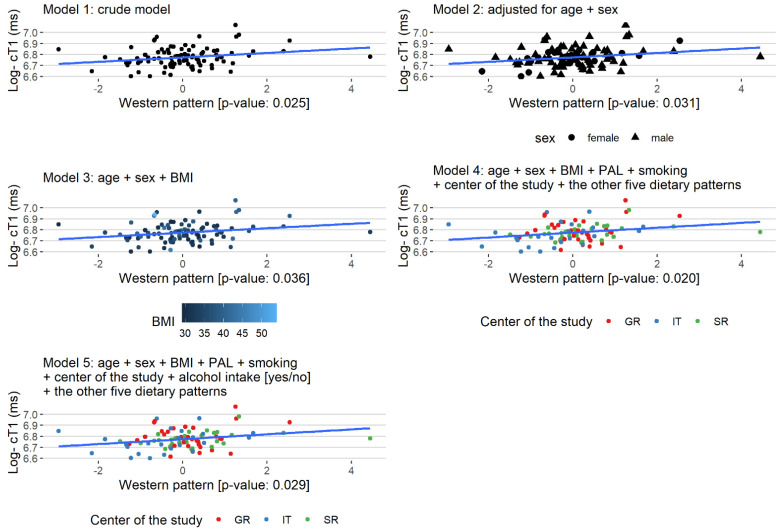
Regression plots of the statistically significant association of the “Western” pattern with Log- cT1 (ms) for the Models 1–5.

The associations of the dietary pattern with the MRI parameters are presented in Table S4 and Table 3. The “Western” pattern was associated with increased values of log-cT1 in Model 1 (beta: 0.020, *p*-value: 0.025). This effect remained significant in all models, even after adjusting for age, sex, BMI, PAL, smoking, the center of the study, and the other five dietary patterns (beta: 0.024, *p*-value: 0.020) (Figure 1). The medium tertile of the “Low-Fat Dairy and Poultry” pattern was associated with lower values of: log-cT1 in Model 1 (beta: −0.047, *p*-value: 0.038) and Model 4 (beta: −0.052, *p*-value: 0.046)); log-PDFF in all models (Model 1 (beta: −0.459, *p*-value: 0.011), Model 2 (beta: −0.392, *p*-value: 0.029), Model 3 (beta: −0.387, *p*-value: 0.032), Model 4 (beta: −0.448, *p*-value: 0.030), and Model 5 (beta: −0.46, *p*-value: 0.027]; LIF in Model 4 (beta: −0.408, *p*-value: 0.025) and Model 5 (beta: −0.412, *p*-value: 0.025) compared to the lowest tertile. No significant associations were found between the other patterns and observed MRI parameters.

## 4. Discussion

The present study examined the association of six dietary patterns of “High-Sugar”, “Prudent”, “Western”, “High-Fat and Salt”, “Plant-Based”, and “Low-Fat Dairy and Poultry” in MAST4HEALTH patients with MRI parameters related to NAFLD progression.

For the first time, the “Western” pattern, which consists mostly of a high intake of refined grains, red meat, and fast food, was associated with increased values of cT1, which is strongly correlated with disease activity [36] and the fibrosis stage [37]. This association was independent of age, sex, BMI, PAL, smoking, center of the study, and the other five dietary patterns. Previous research supports our findings. For example, refined grains are known to rapidly increase insulin and glucose levels in the blood, which are known to contribute to insulin resistance (IR), diabetes, and obesity [38]. Additionally, the rate of de novo production and the acceleration of fat in liver cells are enhanced by increased blood sugar and hyperinsulinemia due to hepatic IR [39]. A high glycemic index diet stimulates the accumulation of fat in the liver cells, leading to hepatic steatosis [39,40]. Moreover, a high intake of red and/or processed meat and fast food has been linked to NAFLD [41,42]. In a recent meta-analysis, individuals who consume more red meat and soft drinks exhibit a significantly increased likelihood of NAFLD [43]. Similarly, dietary patterns containing high levels of red meat and refined grains, as well as high-fat dairy and processed foods, could significantly increase NAFLD by 50% [29]. Saturated fatty acids (SFA) and trans-fatty acids (FA) possibly influence steatosis of hepatic cells by chylomicron intake after consumption of fatty foods [44]. It is worth noting that a recent study in NAFLD patients demonstrated that the heterocyclic amines (HCAs) produced by high temperatures for an extended period of cooking meat, were associated with IR and were found to be hazardous to health [41]. 

Furthermore, the medium tertile of the “Low-Fat Dairy and Poultry” pattern was associated with lower values of cT1, PDFF, and LIF, indicating an inverse relationship with NAFLD. This is not surprising, as high protein intake has been linked with mobilization and a decrease in liver fat. In mice fed with a choline-deficient high-fat diet, which induces NASH, branched-chain amino acids supplementation alleviated hepatic steatosis and liver injury associated with NASH by suppressing the expression of FAS gene and its protein levels [45]. Additionally, whey protein supplementation significantly improved hepatic steatosis and plasma lipid profiles in obese non-diabetic patients compared with an ad libitum diet [46]. In the prospective study of type 2 diabetes patients, Markova et al. [47] found that diets rich in protein and low in fat dramatically decreased liver fat regardless of body weight, as well as indicators of insulin resistance and hepatic necroinflammation. This impact was linked to a decrease in lipolysis and lipogenic indices. 

Although the “Plant-Based” pattern, including whole grains, pulses, and nuts, had no association with MRI parameters, the group with the lower adherence in this pattern exhibited decreased AST/ALT ratio compared to the group of medium adherence. Previous results [48] have shown that the consumption of whole grains improved the levels of liver enzymes and hepatic steatosis in NAFLD patients. In addition, a higher intake of pulses has been related to a decreased risk of NAFLD [49]. A meta-analysis demonstrated that a higher nut intake was negatively associated with NAFLD [43]. Several studies have confirmed a clear connection between nut consumption and reduced levels of inflammation, IR, oxidative stress, and metabolic syndrome, all of which have been implicated in NAFLD progression [50,51,52,53,54,55]. Regarding AST/ALT ratio, it is considered an indicator of liver disease progression and its increase is associated with NAFLD [56]. In line with our findings, in the manuscript published by Tzima et al. [57], the AST/ALT ratio was positively correlated with the Mediterranean Diet, which is considered an established plant-based diet.

The “High-Sugar” pattern is characterized by a high intake of carbohydrate/sugar, which promotes de novo fatty acid production in the liver [38]. Foods with a high glycemic index induce hepatic steatosis, particularly in insulin-resistant individuals [40]. Research findings also highlight the link between a high carbohydrate/sugar pattern and NAFLD in women [58]. However, in the current study, we detected no statistically significant link between the “High-Sugar” pattern consumption and MRI parameters. Interestingly, another research group found no association between fructose intake and NAFLD or risk of hepatic fibrosis; however, in this study, underreporting of sugar intake due to overweight/obesity and diabetes might be the reason for the lack of association [26].

To the best of our knowledge, this is the first study to examine the association of different dietary patterns with NAFLD parameters assessed by MRI-derived measurements of liver fat content, inflammation, and fibrosis. In the analysis, potential confounding variables were identified and for which, adjusted.

There are certain limitations to our study that should be considered. One of this study’s weaknesses was the number and heterogeneity (different origin) of the sample, which was compensated by the strict criteria that were used in the PCA analysis, as well as by the adjustment of the center of the study in the regression Models 4 and 5. Moreover, as participants were obese and diagnosed with NAFLD, underreporting in certain food items may have occurred. In addition, recall bias is possible due to the questionnaire’s self-reporting character. Another significant restriction is that PCA analysis of dietary data incorporates approaches that need subjectivity, such as grouping foods and determining the number of components. Moreover, as a cross-sectional analysis, this study explored the possible associations of dietary patterns with the presence of NAFLD, which does not necessarily indicate that dietary interventions will influence NAFLD severity. When *p* < 0.05 the results are significant, however, there is still a 1/20 chance that this is inaccurate; thus, the replication of our findings in bigger cohorts in future is needed.

## 5. Conclusions

The current study in MAST4HEALTH NAFLD patients indicated that a “Western” dietary pattern with refined grains, red meat, and fast food was positively associated with the MRI marker of liver inflammation and fibrosis. In contrast, a “Low-Fat Dairy and Poultry” pattern was negatively associated with these parameters. Upon validation by future larger cohort studies, the results may assist clinicians to inform people at risk of, or with, NAFLD, of healthy dietary choices.

## Figures and Tables

**Table 1:** Comparison of demographic, anthropometric, lifestyle, MRI, and biochemical parameters in the three centers (Greece: GR, Italy: IT and Serbia: SR) of the study.

	Center of the Study			
Variables	GR (*N*: 38)	IT (*N*: 30)	SR (*N*: 29)	*p*-value
Age ***	51.5 (14.5)	47.5 (12.75)	47 (20)	0.352
Sex (F|M)	11|27	9|21	9|20	0.983
Smoking (Yes|No)	12|25	5|25	4|25	0.136
BMI ***	36.25 (7.12) † ꭅ	32.22 (3.74) †	32.19 (4.31) ꭅ	0.003
PAL (total) ***	1463.25 (1479.22) ꭅ	1188 (1179) ‡	3366.75 (6295.5) ꭅ‡	0.007
FindRisk Score ***	12.5 (4)	13 (4)	14 (6)	0.641
cT1 (ms) ***	875.82 (107.11)	843.83 (106.13)	867.18 (67.56)	0.542
PDFF (%) ***	12.52 (11.09)	15.84 (18.73)	15.73 (11.96)	0.562
LIF *	2.49 (± 1.04) †	2.01 (± 0.97) †	2.26 (±0.54)	0.019
AST (IU/L) ***	20 (7.75) †	27 (11) †	22 (13.5)	0.012
ALT (IU/L) ***	26 (16) †	45 (45) †	35 (18)	0.004
AST/ALT ratio ***	0.71 (0.26)	0.66 (0.29)	0.66 (0.27)	0.204
γ-gt (U/L) ***	30 (13)	41.5 (42.75)	33 (32)	0.175
Total cholesterol (mg/dL) ***	187.5 (42.25)	198.5 (32.5)	203 (71.1)	0.176
HDL (mg/dL) ***	45 (12.75)	45 (11.5)	37.9 (12.4)	0.096
LDL (mg/dL) ***	114 (24)	121 (37.3)	130.7 (58.7)	0.072
Triglycerides (mg/dl) ***	133 (65.5)	132.5 (103)	147 (113.4)	0.534
Glucose (mg/dL) ***	104 (13)	102 (11.25)	99 (14.4)	0.284
120 min-OGTT Glucose (mg/dL) ***	106 (39)ꭅ	116 (41.5) ‡	144 (54) ‡ꭅ	0.007
HOMA-IR ***	4.64 (3.28)	5.51 (4.29) ‡	3.58 (2.5) ‡	0.018
Insulin (μU/mL) ***	16.9 (10.07)	19.95 (19.05) ‡	14.7 (9) ‡	0.049

Note: The normality assumption was checked using the Shapiro–Wilk test; * parametric variable; *** non parametric variable; parametric quantitative variables are expressed as mean (±standard deviation (SD)), non-parametric quantitative variables as median (interquartile range (IQR)) and categorical variables as numbers; *p*-value was obtained using Kruskal–Wallis with Dunn’s post hoc test or ANOVA with Tukey’s post hoc test for continuous non-parametric and parametric variables, respectively, and the chi-square test for categorical variables; † differences between GR and IT, ‡ differences between ΙΤ and SR, ꭅ differences between GR and SR; PAL: physical activity level; FindRisk Score: Finnish diabetic risk score; cT1: included iron-corrected; proton density fat fraction (PDFF); liver inflammation fibrosis score (LIF); AST: aspartate transaminase; ALT: alanine transaminase; AST/ALT ratio: AST to ALT ratio; γ-GT: γ-glutamyltransferase; HDL: high-density lipoprotein; LDL: low-density lipoprotein; HOMA-IR: homeostatic model assessment of insulin resistance.

**Table 2:** Principal component analysis’ factor loadings for the 25 food groups.

Food Groups	High-Sugar Pattern	Prudent Pattern	Western Pattern	High-Fat and Salt Pattern	Plant-Based Pattern	Low-Fat Dairy and Poultry Pattern
Pies	0.77					
Dried fruits	0.74					
Processed meat	0.71					
Fruit juice	0.63					
Sweets	0.59					
Fruits	0.45	0.43				
Sea-food		0.88				
Fish		0.69				
Eggs		0.59				
Vegetable fat		0.58				
Vegetables	0.48	0.53				
Coffee and tea		0.47				
Sauces				0.7		
Dairy (high-fat)				0.57	0.39	
Soft drinks			0.36	0.57		
Animal and hydrogenated fats				0.54		
Salty snacks				0.35		
Refined grains		0.39	0.61			
Red meat		0.44	0.59			
Fast food	0.37		0.53			
Whole grains					0.7	
Pulses					0.68	
Nuts					0.56	0.41
Dairy (low-fat)						0.67
Poultry						0.64
Percent (%) variance explained by each pattern	14%	13%	8%	8%	7%	6%

**Table 3:** The associations of the “Western” and “Low-Fat Dairy and Poultry” patterns with the MRI parameters in the MAST4HEALTH obese and NAFLD patients.

	Western Pattern					Western Pattern	
	Low	Medium		High			
Variables		Beta (SE)	*p*-value	Beta (SE)	*p*-value	Beta (SE)	*p*-value
Log- cT1 (ms)							
Model 1	Ref.	−0.003 (0.022)	0.874	0.037 (0.022)	0.104	0.020 (0.009)	0.025
Model 2	Ref.	−0.004 (0.022)	0.842	0.035 (0.023)	0.134	0.020 (0.009)	0.031
Model 3	Ref.	−0.007 (0.021)	0.762	0.036 (0.022)	0.108	0.019 (0.009)	0.036
Model 4	Ref.	−0.003 (0.025)	0.905	0.038 (0.027)	0.173	0.024 (0.010)	0.020
Model 5	Ref.	−0.003 (0.025)	0.896	0.035 (0.028)	0.211	0.024 (0.011)	0.029
Log-PDFF (%)							
Model 1	Ref.	−0.236 (0.180)	0.192	−0.059 (0.181)	0.743	0.041 (0.074)	0.581
Model 2	Ref.	−0.260 (0.176)	0.143	−0.087 (0.179)	0.629	0.033 (0.072)	0.649
Model 3	Ref.	−0.268 (0.176)	0.132	−0.088 (0.180)	0.625	0.030 (0.073)	0.679
Model 4	Ref.	−0.299 (0.200)	0.141	−0.090 (0.215)	0.678	0.072 (0.085)	0.401
Model 5	Ref	−0.296 (0.202)	0.147	−0.08 (0.22)	0.719	0.081 (0.088)	0.359
LIF							
Model 1	Ref.	0.023 (0.156)	0.882	0.249 (0.160)	0.123	0.121 (0.063)	0.059
Model 2	Ref.	0.020 (0.158)	0.897	0.243 (0.164)	0.142	0.119 (0.064)	0.067
Model 3	Ref.	0.005 (0.153)	0.974	0.254 (0.158)	0.113	0.111 (0.062)	0.078
Model 4	Ref.	−0.049 (0.174)	0.777	0.138 (0.192)	0.473	0.105 (0.073)	0.154
Model 5	Ref	−0.05 (0.175)	0.775	0.13 (0.196)	0.508	0.103 (0.075)	0.175
	**Low-Fat Dairy and Poultry Pattern**					**Low-Fat Dairy and Poultry Pattern**	
	**Low**	**Medium**		**High**			
**Variables**		**Beta (SE)**	***p*-value**	**Beta (SE)**	***p*-value**	**Beta (SE)**	***p*-value**
Log- cT1 (ms)							
Model 1	Ref.	−0.047 (0.022)	0.038	−0.021 (0.022)	0.343	−0.008 (0.009)	0.378
Model 2	Ref.	−0.045 (0.023)	0.051	−0.020 (0.023)	0.380	−0.008 (0.009)	0.406
Model 3	Ref.	−0.043 (0.022)	0.059	−0.018 (0.022)	0.416	−0.011 (0.009)	0.221
Model 4	Ref.	−0.052 (0.025)	0.046	−0.016 (0.023)	0.499	−0.012 (0.010)	0.228
Model 5	Ref	−0.051 (0.026)	0.051	−0.016 (0.023)	0.503	−0.011 (0.010)	0.239
Log-PDFF (%)							
Model 1	Ref.	−0.459 (0.177)	0.011	−0.209 (0.178)	0.243	−0.042 (0.074)	0.574
Model 2	Ref.	−0.392 (0.177)	0.029	−0.115 (0.181)	0.525	−0.017 (0.073)	0.820
Model 3	Ref.	−0.387 (0.178)	0.032	−0.110 (0.181)	0.547	−0.023 (0.074)	0.757
Model 4	Ref.	−0.448 (0.202)	0.030	−0.078 (0.191)	0.686	−0.023 (0.079)	0.768
Model 5	Ref	−0.46 (0.204)	0.027	−0.076 (0.192)	0.695	−0.025 (0.08)	0.756
LIF							
Model 1	Ref.	−0.294 (0.158)	0.066	−0.125 (0.157)	0.426	−0.038 (0.064)	0.554
Model 2	Ref.	−0.290 (0.163)	0.079	−0.123 (0.163)	0.451	−0.036 (0.066)	0.583
Model 3	Ref.	−0.270 (0.159)	0.092	−0.109 (0.158)	0.494	−0.061 (0.064)	0.342
Model 4	Ref.	−0.408 (0.178)	0.025	−0.125 (0.163)	0.444	−0.071 (0.067)	0.297
Model 5	Ref	−0.412 (0.18)	0.025	−0.126 (0.164)	0.446	−0.07 (0.068)	0.304

The cT1 (ms) and PDFF (%) were log-transformed due to the skewness of the distribution. Four adjustment sets were considered: Model 1: crude model; Model 2: adjusted for age + sex; Model 3: adjusted for Model 2 + BMI; Model 4: adjusted for Model 3 + PAL + smoking + center of the study + the other five dietary patterns; Model 5: adjusted for Model 4 + alcohol intake [yes/no]. A *P* value < 0.05 was considered significant in all tests. Ref: Reference (the low tertile of each dietary pattern was used as a reference group). Beta: beta coefficient. SE: standard error.

## Data Availability

Data available on request due to restrictions, e.g., privacy or ethical. The data presented in this study are available on request from the corresponding author. The data are not publicly available due to privacy/ethical restrictions on the data provided by the volunteers.

## Supplementary Materials

The following are available online at https://www.mdpi.com/article/10.3390/ijerph19020971/s1. Table S1, 66 food items were categorized into 25 food groups. Table S2: The comparison of demographic, anthropometric, lifestyle, MRI, and biochemical parameters in different levels of the dietary patterns. Table S3: Daily energy and nutrients intake in different levels of the dietary patterns. Table S4: The associations of the “High-sugar”, “Prudent”, “High-Fat and Salt”, and “Plant-Based” patterns with the MRI parameters in the MAST4HEALTH obese and NAFLD patients.
