# Supplementary material for: Association of Dietary Patterns with MRI Markers of Hepatic Inflammation and Fibrosis in the MAST4HEALTH Study
Source: Int J Environ Res Public Health. 2022 Jan 16;19(2):971. doi: 10.3390/ijerph19020971 (PMC8775335; doi:10.3390/ijerph19020971)
Supplement: Supplementary file 1 [file ijerph-19-00971-s001.zip › ijerph-1506047-supplementary.pdf]

Supplementary Material

Table S1. 66 food items were categorized into 25 food groups.

	Food Groups	Food Items (66)
**1**	Vegetables ǀ cooked mixed vegetables	Tomato/cucumber/carrot/pepper ǀ Broccoli/cauliflower/courgetti ǀ Lettuce/cabbage/spinach/rocket ǀ Greens/celery/spinach ǀ Spinach-rice/ cabbage-rice ǀ Petit pois (peas) / green beans / okra /artichoke
**2**	Fruits	Orange ǀ Apple/pear ǀ Other winter-fruits ǀ banana ǀ Other summer – fruits
**3**	Fruit juice	
**4**	Dried fruits	
**5**	Eggs	
**6**	Fish	small fish ǀ large fish
**7**	Sea-food	Sea-food (octopus, sleeve-fish, prawns)
**8**	Pulses	Pulses (lentils, beans, chickpeas)
**9**	Nuts	
**10**	Pies	Home made pies (e.g. Cheese-pie, spinach-pie) ǀ Pies
**11**	Fast Food	Toasted sandwich/sandwich ǀ burger-bread ǀ French fried potatoes
**12**	Salty Snacks	Chips/pop-corn ǀ crisp bread
**13**	Sweets	Sweets made in tray ǀ Sweet preserves/stewed fruit/fruit – jelly ǀ Gateau/tart ǀ Croissant/gofer/cake/biscuits ǀ chocolate ǀ Honey/marmalade/sugar ǀ Cereal/cereal bars
**14**	Sauces	Mayonnaise/sauce ǀ Light mayonnaise/light sauce
**15**	Vegetable Fat	seed oil, olive oil ǀ olives
**16**	Animal & Hydrogenated Fats	Butter ǀ margarine
**17**	Poultry	Chicken/turkey (all kind)
**18**	Red Meat	Veal (steak, filet) ǀ Burger/ meat balls/ minced-meat ǀ Pork (steak, filet) ǀ Lamb/goat/game/ lamb-chops ǀ Pastitsio/moysakas/papoytsakia
**19**	Processed Meat	Sausage / bacon ǀ Light/no fat cold sliced meats ǀ Cold sliced meats

**20**	Dairy (Low-fat)	Low fat milk/yogurt ǀ Low fat cheese (light/ cottage)
**21**	Dairy (High-fat)	High fat milk/yogurt ǀ Yellow cheese/cream cheese ǀ White cheese (e.g. feta cheese) ǀ Ice-cream/milk-shake/cream/rice pudding
**22**	Refined grains	White bread/toast ǀ white rice ǀ Pasta/ pearl barley ǀ Potatoes boiled/baked/mashed
**23**	Whole grains	Whole meal bread/rusk ǀ brown rice ǀ Whole meal pasta
**24**	Soft drinks	Soft drinks ǀ Light soft drinks
**25**	Coffee and Tea	Coffee, Tea/other teas

Table S2. The comparison of demographic, anthropometric, lifestyle, MRI and biochemical parameters in different levels of the dietary patterns.

	**High-Sugar pattern**				**Prudent pattern**			
**Variables**	Low	Medium	High	p-value	Low	Medium	High	p-value
**age** ***	49 (14)	51 (18)	48 (13)	0.9767	46.5 (15.25)	49 (17.5)	49 (10)	0.3838
**sex (FǀM)**	10ǀ22	10ǀ22	9ǀ24	0.9211	8ǀ24	12ǀ20	9ǀ24	0.5073
**center of the study (GRǀITǀSR)**	13ǀ12ǀ7	13ǀ12ǀ7	12ǀ6ǀ15	0.1481	13ǀ5ǀ14	14ǀ9ǀ9	11ǀ16ǀ6	**0.04265**
**smoking (YesǀNo)**	4ǀ27	8ǀ24	9ǀ24	0.3319	9ǀ23	6ǀ26	6ǀ26	0.5778
**BMI** ***	32.3 (4.49)	34.01 (3.61)	34.43 (7.83)	0.3385	32.03 (4.06)	33.56 (7.02)	34.72 (6.72)	0.1941
**PAL (total)** ***	1406.25 (1419)	1775.75 (3716.25)	1748 (3527.5)	0.419	1694.5 (6962)	1658.25 (2032.75)	1552.5 (1742.62)	0.459
**FindRisk Score** ***	13 (4)	14 (4.5)	13 (4)	0.9642	13 (4)	12 (4)	14 (5)	0.3456
**cT1 (ms)** ***	882.23 (88.8)	862.64 (68.07)	868.6 (107.06)	0.7391	864.62 (93.81)	874.71 (93.61)	877.42 (68.74)	0.8671
**PDFF (%)** ***	12.4 (13.82)	14.05 (14.88)	16.27 (15.14)	0.5405	15.18 (16.33)	11.14 (10.67)	15.68 (15.79)	0.3428
**LIF** *	2.26 (±0.7)	2.2 (±0.6)	2.32 (±0.59)	0.741	2.32 (±0.6)	2.19 (±0.62)	2.27 (±0.67)	0.701
**AST (IU/L)*****	23 (7.25)	24.5 (11)	22 (11)	0.7911	22.5 (9.75)	22 (10.75)	23.5 (11.25)	0.4678

**ALT (IU/L)*** **	28.5 (28)	36 (22.25)	33.5 (19.5)	0.8233	28.5 (20.5)	28 (26.25)	37 (20.75)	0.1741
**AST/ALT ratio *** **	0.7 (0.24)	0.68 (0.33)	0.66 (0.18)	0.7347	0.73 (0.22)	0.68 (0.34)	0.66 (0.24)	0.1738
**γ-gt (U/L) *** **	33 (34.5)	39.5 (43)	30 (13)	0.1291	33.5 (32.25)	31 (16.5)	33 (37)	0.4347
**Total cholesterol (mg/dL)*** **	196.1 (45)	200.5 (44.75)	194 (48.4)	0.4455	196 (48.7)	196.1 (37.62)	194 (47)	0.8564
**HDL (mg/dL)*** **	46 (13.85)	42.45 (12.47)	41 (12.7)	0.3645	42 (10.77)	45.5 (13.85)	44 (16)	0.2515
**LDL (mg/dL)*** **	121 (32.5)	125.5 (43.3)	114 (42.8)	0.2581	125 (46.05)	120 (24)	113.3 (33)	0.5244
**triglycerides (mg/dl)*** **	130 (87.67)	137.5 (119.9)	132 (72.9)	0.7201	143 (64.42)	131 (98.03)	130 (114)	0.5312
**glucose (mg/dL) *** **	100 (12)	102.5 (13.8)	100.8 (17)	0.7956	97.2 (13.4)	104.4 (11.4)	100.9 (12.25)	0.1628
**120 min-OGTT glucose (mg/dL) *** **	129 (47.9)	108 (44.2)	128.7 (72.2)	0.2391	125.1 (65.7)	111 (40.05)	128.5 (62.2)	0.6484
**HOMA-IR*** **	4.45 (2.87)	4.27 (3.14)	4.86 (3.23)	0.7515	4.2 (2.57)	4.58 (2.94)	4.53 (4.71)	0.8925
**Insulin (μU/mL)*** **	15.8 (9.65)	17 (8.25)	18.2 (11.55)	0.8129	16.2 (8.12)	17.7 (9.1)	16.45 (16.8)	0.9895

Note: The normality assumption was checked using the Shapiro-Wilk test.; * parametric variable, *** non parametric variable.; Parametric quantitative variables are expressed as mean (± standard deviation (SD)), non-parametric quantitative variables as median (interquartile range (IQR)) and categorical variables as numbers.; P value was obtained using Kruskal-Wallis with Dunn's post-hoc test or ANOVA with Tukey's post-hoc test for continuous non parametric and parametric variables, respectively and chi-square test for categorical variables; †Differences between low and high tertile, ‡Differences between medium and high tertile, ﬞ Differences between low and medium tertile; PAL: physical activity level; FindRisk Score: Finnish Diabetic Risk Score; cT1: included iron-corrected; proton density fat fraction (PDFF); Liver Inflammation Fibrosis score (LIF); AST: aspartate transaminase; ALT: alanine transaminase; AST/ALT ratio: AST to ALT ratio; γ-GT: γ-glutamyltransferase; HDL: High-density lipoprotein; LDL: Low-density lipoprotein; HOMA-IR: homeostatic model assessment of insulin resistance

Table S2. Cont.

	**Western pattern**				**High-Fat And Salt pattern**			
**Variables**	Low	Medium	High	p-value	Low	Medium	High	p-value
**age *** **	51.5 (9.5)	48 (16)	44.5 (14)	0.1716	49 (13)	53 (16.5)	46 (12)	0.2832
**sex (F|M)**	11|21	10|23	8|24	0.7136	11|22	8|23	10|23	0.8041

**center of the study (GR I IT I SR)**	9 I 17 I 6	14 I 7 I 12	15 I 6 I 11	**0.02424**	11 I 12 I 10	9 I 11 I 11	18 I 7 I 8	0.2578
**smoking (Yes I No)**	8 I 24	7 I 25	6 I 26	0.8329	5 I 27	9 I 22	7 I 26	0.4341
**BMI *****	32.51 (6.18)	34.21 (6.17)	33.08 (4.63)	0.7394	33.78 (4.41)	33.33 (7.16)	32.98 (6.89)	0.8633
**PAL (total) *****	1323 (1114.12)	1701 (2378.25)	2310 (6326.75)	0.1406	1959 (2837.25)	1386 (1586.5)	1813.5 (5213.62)	0.5262
**FindRisk Score *****	14 (5.5)	12 (3)	13.5 (4)	0.09009	13 (6)	14 (4)	12 (3.25)	0.3044
**cT1 (ms) *****	862.07 (82.85)	870.02 (69.86)	884.34 (83.61)	0.2503	855.42 (73.46)	876.57 (107.32)	874.56 (93.91)	0.6181
**PDFF (%) *****	17.13 (13.28)	12.34 (9.76)	11.07 (16.5)	0.3005	12.4 (13.61)	15.14 (13.61)	14.88 (14.64)	0.6684
**LIF ***	2.17 (±0.71)	2.2 (±0.58)	2.42 (±0.57)	0.232	2.14 (±0.62)	2.34 (±0.66)	2.31 (±0.61)	0.403
**AST (IU/L)*****	25 (12.5)	21 (6)	24 (12.5)	0.05509	24.5 (11.25)	23 (9.5)	22 (11)	0.4289
**ALT (IU/L)*****	42 (32)ሰ	27 (11)ሰ ‡	36 (24) ‡	**0.02449**	27 (24.25)	36 (26.5)	30 (23.5)	0.5363
**AST/ALT ratio *****	0.64 (0.23)	0.78 (0.25)	0.69 (0.28)	0.1432	0.7 (0.3)	0.66 (0.21)	0.7 (0.27)	0.6862
**γ-gt (U/L) *****	36 (45)	31 (16.75)	32.5 (52.5)	0.1003	41 (32)	33 (17.5)	30.5 (25.75)	0.7913
**Total cholesterol (mg/dL)*****	198.5 (29.5)	188.3 (52)	192.5 (44.5)	0.6382	202 (43)	198 (46)	187 (33)	0.6733
**HDL (mg/dL)*****	48.5 (15.5)	42 (11.3)	41 (11.45)	0.2686	43 (12)	44 (17.5)	41 (13.1)	0.9815
**LDL (mg/dL)*****	122.95 (28)	114.1 (41)	125 (42.3)	0.4991	125.5 (44.25)	128.8 (49.2)	117 (19)	0.6083
**triglycerides (mg/dl)*****	131.5 (93.75)	138 (107)	133.45 (69.68)	0.7657	134 (114)	129.3 (81.9)	136 (98)	0.8138
**glucose (mg/dL) *****	105 (14)	99.5 (12.9)	98 (12.8)	0.1106	100.9 (10.1)	103.7 (22.7)	100.8 (12)	0.8742
**120 min-OGTT glucose (mg/dL) *****	121 (50.4)	117 (43.4)	129.6 (65.6)	0.7644	111 (43.2)	132.5 (62)	119 (46.4)	0.5656
**HOMA-IR*****	4.93 (4.07)	4.08 (2.53)	4.57 (3.12)	0.3886	4.08 (2.93)	5.03 (3.44)	4.53 (2.29)	0.8636
**Insulin (μU/mL)*****	17.55 (14.35)	15.8 (7.4)	18 (9.27)	0.6377	14.1 (12.85)	17.4 (8.45)	18.55 (8.08)	0.5436

Note: The normality assumption was checked using the Shapiro-Wilk test.; * parametric variable, *** non parametric variable.; Parametric quantitative variables are expressed as mean (± standard deviation (SD)), non-parametric quantitative variables as median (interquartile range (IQR)) and categorical variables as numbers.; P value was obtained using Kruskal-Wallis with Dunn's post-hoc test or ANOVA with Tukey's post-hoc test for continuous non parametric and parametric variables, respectively and chi-square test for categorical variables; †Differences between low and high tertile, ‡Differences between medium and high tertile, ሰ Differences between low and medium tertile; PAL: physical activity level; FindRisk Score: Finnish Diabetic Risk Score; cT1: included iron-corrected; proton density fat fraction (PDFF); Liver Inflammation

Fibrosis score (LIF); AST: aspartate transaminase; ALT: alanine transaminase; AST/ALT ratio: AST to ALT ratio; γ-GT: γ-glutamyltransferase; HDL: High-density lipoprotein; LDL: Low-density lipoprotein; HOMA-IR: homeostatic model assessment of insulin resistance

Table S2. Cont.

	Plant-based pattern				Low-Fat Dairy and Poultry pattern			
**Variables**	Low	Medium	High	p-value	Low	Medium	High	p-value
**age *****	47 (17)	46 (15.5)	50 (9)	0.3569	46.5 (11.25)	50 (17)	50.5 (17.25)	0.5954
**sex (F|M)**	6|27	14|17	9|24	0.05739	5|27	11|22	13|19	0.07993
**center of the study (GR|IT|SR)**	8|17|8	14|7|10	16|6|11	**0.03439**	12|11|9	13|9|11	13|10|9	0.973
**smoking (Yes|No)**	8|25	8|23	5|27	0.5712	9|23	4|28	8|24	0.278
**BMI *****	32.04 (4.04)	33.28 (6.41)	34.72 (7.83)	0.1304	34.32 (6.62)	32.93 (7.74)	32.91 (4.49)	0.8992
**PAL (total) *****	1632 (4308)	1635 (2766)	1688.25 (1659.68)	0.9832	1362 (1553.62)	2185.5 (3231.75)	1748 (4725.75)	0.2665
**FindRisk Score *****	13 (3.25)	13 (4)	14 (4)	0.9089	13 (4)	14 (7)	13 (4.25)	0.6292
**cT1 (ms) *****	870.02 (108.71)	849.73 (59.3)	878.88 (88.62)	0.1643	875.82 (115.07)	843.86 (85.86)	874.04 (68.02)	0.2274
**PDFF (%) *****	15.82 (14.64)	10.94 (12.13)	16.55 (14.6)	0.2453	16.66 (18.91)ለ	11.02 (11.99)ለ	14.16 (13.23)	**0.04452**
**LIF ***	2.24 (±0.68)	2.11 (±0.52)	2.42 (±0.64)	0.164	2.4 (±0.63)	2.11 (±0.64)	2.27 (±0.6)	0.181
**AST (IU/L)*****	25 (14.5)ለ	19 (7.5)ለ ‡	24 (12) ‡	**0.04727**	25 (6.25)	19 (12)	24 (11)	0.1959
**ALT (IU/L)*****	41 (31)ለ	26 (17)ለ ‡	36 (21) ‡	**0.0138**	38 (16.25)	27 (15)	30 (21)	0.06961
**AST/ALT ratio *****	0.65 (0.25)ለ	0.83 (0.31)ለ	0.66 (0.17)	**0.04029**	0.64 (0.21)	0.7 (0.28)	0.72 (0.24)	0.1598
**γ-gt (U/L) *****	37 (28)	30 (19)	32 (32)	0.2255	30.5 (25.75)	31.5 (20.5)	36.5 (46.75)	0.4328
**Total cholesterol (mg/dL)*****	197 (40)	204 (40)	188.3 (55.7)	0.1722	186.5 (28.8)	199 (46.8)	200 (56.5)	0.2723
**HDL (mg/dL)*****	41 (13)	45 (12.15)	44 (14)	0.3598	41 (10.07)	45 (19.7)	46.5 (13.5)	0.2692
**LDL (mg/dL)*****	118 (39)	126 (40.3)	116.05 (42.55)	0.3144	117 (27.7)	124.9 (56)	121 (37.5)	0.4185
**triglycerides (mg/dl)*****	159 (91.3)	132.9 (111)	130 (87)	0.8775	135 (70.58)	121 (101.3)	141.5 (111)	0.5033
**glucose (mg/dL) *****	97 (18)	104 (12)	101.4 (10.85)	0.7956	102 (14)	100.8 (14.9)	100.4 (13.15)	0.7549

**120 min-OGTT glucose (mg/dL) *****	129.3 (33.25)	106 (38.7)	132.9 (74.9)	0.1423	129.6 (44.8)	129 (64)	106.5 (36.15)	0.3122
**HOMA-IR*****	5.03 (3.81)	4.4 (2.92)	4.08 (2.7)	0.3624	5.59 (4.15)	4.65 (2.45)	4.08 (2.11)	0.09498
**Insulin (µU/mL)*****	18.3 (14.5)	15.7 (9.47)	14.6 (7.25)	0.229	19.4 (14.6)	14.8 (9.25)	16.1 (8.05)	0.06205

Note: The normality assumption was checked using the Shapiro-Wilk test.; * parametric variable, *** non parametric variable.; Parametric quantitative variables are expressed as mean (± standard deviation (SD)), non-parametric quantitative variables as median (interquartile range (IQR)) and categorical variables as numbers.; P value was obtained using Kruskal-Wallis with Dunn's post-hoc test or ANOVA with Tukey's post-hoc test for continuous non parametric and parametric variables, respectively and chi-square test for categorical variables; †Differences between low and high tertile, ‡Differences between medium and high tertile, ለ Differences between low and medium tertile; PAL: physical activity level; FindRisk Score: Finnish Diabetic Risk Score; cT1: included iron-corrected; proton density fat fraction (PDFF); Liver Inflammation Fibrosis score (LIF); AST: aspartate transaminase; ALT: alanine transaminase; AST/ALT ratio: AST to ALT ratio; γ-GT: γ-glutamyltransferase; HDL: High-density lipoprotein; LDL: Low-density lipoprotein; HOMA-IR: homeostatic model assessment of insulin resistance

Table S3. Daily energy and nutrients intake in different levels of the dietary patterns.

	**High-Sugar pattern**			
**Nutrients**	**Low**	**Medium**	**High**	**p-value**
protein (g) ***	74.46 (51.56)	83.68 (47.38)	99.24 (41.25)	0.1947
Total fat (g) ***	86.39 (43.48)	90.19 (42.14)	101.7 (43.35)	0.205
carbohydrate (g) *	161.01 (± 51.96)	194.84 (± 55.66)	185.52 (± 68.74)	0.0865
kilocalories (kcal) ***	1762.23 (1124.94)	1938.4 (683.7)	2109.59 (598.98)	0.2411
Total sugar (g) ***	39.39 (31.81) ለ	63.89 (33.38) ለ	57.86 (31.91)	**0.01003**
glucose (g) ***	4.51 (6.48)†ለ	10.81 (8.5) ለ	8.84 (8.14)†	**0.008712**
sucrose (g) ***	5.34 (13.28)	10.23 (16)	9.46 (9.41)	0.2254
maltose (g) ***	1.12 (1.14)	1.01 (1.22)	1.14 (0.75)	0.6883
fructose (g) ***	4.87 (7.75)†ለ	12.22 (11.76)ለ	10.84 (13.53)†	**0.005621**
galactose (g) ***	0.04 (0.11)	0.01 (0.09)	0.01 (0.14)	0.6996
lactose (g) ***	4.57 (10.29)	2.64 (6.4)	1.32 (4.8)	0.4118
Amino acids				
alanine (mg) ***	2049.4 (2657.06)	2518.57 (1495.98)	2572.23 (1641.28)	0.6494

arginine (mg) ***	2239.57 (3451.11)	2731.37 (2252.37)	2919.63 (2091.89)	0.5638
aspartic acid (mg) ***	3562.62 (4504.84)	4820.44 (3048.28)	4628.49 (3567.11)	0.5702
cystine (mg) ***	611.6 (689.52)	685.12 (380.53)	732.07 (514.74)	0.8013
glutamic acid (mg) ***	9933.53 (8965.42)	11051.6 (6623.87)	10925.33 (7523.55)	0.8137
glycine (mg) ***	1782.95 (2639.51)	2039.59 (1513.9)	2223.24 (1283.51)	0.6641
histidine (mg) ***	1207.58 (1454.3)	1435.39 (990.82)	1506.43 (1433.53)	0.5898
isoleucine (mg) ***	2056.04 (2316.82)	2274.35 (1243.13)	2290.56 (1818.09)	0.6489
leucine (mg) ***	3483.69 (3754.15)	3993.07 (2138)	4021.84 (3359.46)	0.6523
lysine (mg) ***	2851.52 (3566.25)	3379.38 (2228.43)	3522.08 (3154.59)	0.6288
methionine (mg) ***	993.39 (1262.38)	1101.66 (639.90)	1215.51 (900.52)	0.6342
phenylalanine (mg) ***	1975.25 (2127.38)	2273.54 (1324.89)	2398.32 (1914.1)	0.7037
proline (mg) ***	3099.39 (2236.46)	3599.5 (2315.64)	3151 (2516.46)	0.6852
serine (mg) ***	1908.32 (2052.87)	2330.56 (1631.12)	2379.75 (1994.4)	0.7282
threonine (mg) ***	1740.78 (1986.2)	2029.24 (1088.82)	2028.52 (1463.21)	0.6502
tryptophan (mg) ***	522.83 (584.51)	633.54 (339.65)	610.76 (499.11)	0.693
tyrosine (mg) ***	1391.35 (1663.01)	1677.18 (1082.89)	1766.34 (1562.84)	0.6768
valine (mg) ***	2299.18 (2441.84)	2646.68 (1545.84)	2631.68 (2174.05)	0.6926
Lipids				
cholesterol (mg) ***	157.6 (209.43) †	205.22 (193.1)	259.42 (189.02) †	**0.04234**
SFA (g) *	28.24 (± 11.22)	27.88 (± 12.18)	32.68 (± 11.48)	0.212
Trans-FA (g) ***	0.31 (0.46)	0.26 (0.47)	0.27 (0.59)	0.6067
MUFA (g) ***	38.61 (17.23)	35.98 (20.55)	39.87 (17.46)	0.7139
Oleic acid (g) ***	28.88 (10.26)	30.77 (13.67)	30.25 (16.25)	0.9421
PUFA (g) ***	12.96 (7.76)	12.12 (5.52) ‡	16.39 (11.69) ‡	**0.03617**
Linolenic acid (g) ***	0.81 (0.53)	0.86 (0.61)	1 (0.74)	0.4046
Linoleic acid (g)***	10.76 (5.88)	9.72 (6.88)	12.4 (8.91)	0.1335
DHA (g) ***	0.04 (0.04)	0.04 (0.07)	0.01 (0.03)	0.09175

EPA (g) ***	0.01 (0.02)	0.01 (0.02)	0 (0.01)	0.1289
Total Dietary Fiber (g) ***	15.65 (11.39)	17.55 (11.35)	16.59 (9.62)	0.2215
crude fiber (g) ***	2.41 (2.37)	3.59 (3.64)	2.84 (2.8)	0.1634
insoluble fiber (g) ***	0.35 (1.14)	0.98 (1.24)	0.77 (1.65)	0.07685
soluble fiber (g) ***	0.06 (0.19) †	0.31 (0.54)	0.13 (0.27) †	**0.04174**
Minerals				
zinc (mg) ***	8.11 (5.14)	8.87 (4.6)	9.58 (6.22)	0.2396
copper (mg) ***	0.96 (0.61)	0.96 (0.48)	0.93 (0.42)	0.95
chromium (mg) ***	0.03 (0.04)	0.03 (0.04)	0.04 (0.03)	0.481
selenium (µg) *	110.66 (± 42.99)	104.13 (± 43.77)	115.38 (± 35.98)	0.563
manganese (mg) ***	1.62 (1.68)	2.16 (1.48)	1.96 (1.02)	0.5845
magnesium (mg) ***	241.71 (119.99)	234.41 (135.13)	249.87 (104.47)	0.8628
molybdenum (µg) ***	12.47 (31.73)	13.02 (31.6)	12.18 (10.37)	0.9975
iron (mg) ***	13.89 (8.41)	14.38 (6.03)	14.03 (4.7)	0.8644
iodine (µg) ***	3.94 (6.81)	7.2 (9.32)	9.75 (14.23)	0.6923
calcium (mg) ***	674.79 (378.57)	869.33 (354.02)	790.22 (343.24)	0.3121
fluoride (µg) ***	454.02 (1940.15)	334.08 (2358.13)	316.94 (792.01)	0.9457
phosphorus (mg) ***	1009.6 (678.21)	1089.42 (453.06)	1258.43 (523.39)	0.4431
potassium (mg) ***	2293.44 (885.95)	2181.58 (1010.54)	2605.5 (1082.91)	0.3065
sodium (mg) ***	1714.48 (1066.08)	2225.17 (1153.11)	2251.53 (978.45)	0.07366
Vitamins				
Vitamin A (IU) ***	2199.16 (2776)	2683.28 (3655.17)	4417.28 (8094.89)	0.1459
Vitamin A (RAE) (µg) ***	267.62 (197.25)	322.76 (296.2)	453.02 (517.23)	0.06012
Vitamin A (RE) ***	415.74 (376.5)†	464.88 (545.08)	859.78 (903.88)†	**0.04206**
Vitamin C (mg) ***	40.96 (29.24)	68.51 (107.94)	61.49 (63.94)	0.1084
Vitamin D (µg) ***	2.14 (3.23)	1.94 (3.6)	2.51 (2.02)	0.7293
Vitamin D (IU) ***	79.74 (121.05)	78.29 (130.25)	86.27 (85.04)	0.8984

Vitamin E (mg) ***	0.55 (1.15)	0.82 (1.02)	1.54 (2.09)	0.4508
Vitamin E (IU) ***	0.68 (1.35)	0.78 (1.42)	1.84 (2.6)	0.3868
Vitamin K (µg) ***	67.38 (55.21)	69.22 (59.74)	84.45 (85.15)	0.5982
Cobalamin (Vitamin B12) (µg) ***	3.59 (2.03)	3.4 (2.64)	3.62 (3.11)	0.8848
biotin (µg) ***	10.68 (10.7)	9.93 (10.63)	10.78 (6.15)	0.97
thiamin (mg) ***	1.22 (1.05)	1.41 (0.61)	1.62 (0.71)	0.2215
niacin (mg) ***	20.11 (16.3)	20.38 (11.14)	25.14 (13.13)	0.5791
pantothenic (mg) ***	3.56 (1.97)	3.65 (1.32)	3.69 (3.15)	0.8946
Pyridoxine (Vitamin B6) (mg) ***	1.46 (0.87)	1.53 (0.9)	1.64 (0.9)	0.138
Alpha-Carotene (µg)***	89.2 (237.49)	144.61 (566.66)	275.03 (800.29)	0.1988
Beta-Carotene (µg)***	577.95 (1137.14)	1082.14 (2680.29)	1100.81 (3785.61)	0.269
Beta-Cryptoxanthin (µg) ***	12.9 (114.25)	61.71 (248.95)	36.42 (104.04)	0.1931
Lutein (+ Zeaxanthin) (µg) ***	601.95 (761.61)	1008.71 (1416.83)	837.57 (1484.78)	0.5419
lycopene (µg) ***	1472.39 (2528)	2133.57 (4382.85)	1955.8 (3657.39)	0.9489
Folate (DFE) (µg) ***	284.68 (241.82)	360.39 (210.02)	324.88 (161.16)	0.2311
Folate (Total) (µg) ***	237.09 (164.57)	293.12 (138.3)	262.54 (166.21)	0.2311
riboflavin (mg) ***	1.55 (1.24)	1.59 (0.78)	1.69 (0.89)	0.6396
Alpha-Tocopherol (mg) ***	7.59 (3.86)	7.93 (4.74)	9.39 (5.89)	0.144

Note: The normality assumption was checked using the Shapiro-Wilk test.; * parametric variable, *** non parametric variable.; Parametric quantitative variables are expressed as mean (± standard deviation (SD)) and non-parametric quantitative variables as median (interquartile range (IQR)); P value was obtained using Kruskal-Wallis with Dunn's post-hoc test or ANOVA with Tukey's post-hoc test for continuous non parametric and parametric variables, respectively; †Differences between low and high tertile, ‡Differences between medium and high tertile. ᕱ Differences between low and medium tertile.

Table S3. Cont.

	Prudent pattern			
**Nutrients**	**Low**	**Medium**	**High**	**p-value**
protein (g) ***	99.74 (53.45)	83.68 (39.91)	78.67 (37.05)	0.156
Total fat (g) ***	104.91 (53.35)	101.43 (47.85)	87 (26.31)	0.236
carbohydrate (g) *	194.99 (± 74.62)	182.74 (± 49.39)	165.49 (± 51.73)	0.164
kilocalories (kcal) ***	2133.4 (666.83)	1959.6 (703.03)	1760.76 (649.54)	0.08794
Total sugar (g) ***	60.56 (31.96)	61.63 (30.37)	42.69 (31.11)	0.07937
glucose (g) ***	10.04 (9.38)	8.54 (8.17)	6.68 (7.14)	0.7148
sucrose (g) ***	11.45 (16.96)	9.26 (11.98)	6.43 (9.25)	0.5719
maltose (g) ***	1.11 (1.16)	1.14 (1.29)	1 (0.63)	0.3196
fructose (g) ***	11.16 (15.19)	10.17 (9.58)	8.74 (15.03)	0.9347
galactose (g) ***	0.01 (0.04) ለ	0.09 (0.22) ለ	0.02 (0.06)	**0.03852**
lactose (g) ***	1.72 (6.12)	5.41 (7.46)	0.59 (5.08)	0.06544
Amino acids				
alanine (mg) ***	2694.49 (3252.74)	2629.86 (1644.43)	2118.33 (1391.23)	0.5601
arginine (mg) ***	3092.77 (3194.58)	2978.43 (2352.71)	2345.64 (1943.49)	0.4958
aspartic acid (mg) ***	4644.38 (4690.32)	4873.95 (3780.76)	3701.22 (2541.18)	0.444
cystine (mg) ***	735.99 (659.19)	757.64 (385.9)	608.43 (346.76)	0.5132
glutamic acid (mg) ***	11354.35 (8422.82)	11300.41 (7066.95)	9159.58 (6056.36)	0.3941
glycine (mg) ***	2404.93 (2565.33)	2250 (1614.21)	1800.35 (1061.17)	0.4847
histidine (mg) ***	1547.5 (1903.96)	1579.4 (1226.89)	1236.08 (836.25)	0.5069
isoleucine (mg) ***	2571.15 (2868.49)	2347.62 (1406.95)	2017.95 (1306.99)	0.4975
leucine (mg) ***	4274.06 (4812.98)	3993.07 (2380.7)	3376.28 (2229.53)	0.51
lysine (mg) ***	3798.04 (5118.59)	3600.64 (2773.53)	3087.86 (2160.56)	0.5734
methionine (mg) ***	1318.47 (1664.10)	1162.06 (903.52)	1036.09 (591.04)	0.5354
phenylalanine (mg) ***	2534.96 (2373.44)	2359.57 (1426.72)	1975.25 (1224.36)	0.4595
proline (mg) ***	3441.87 (2497.32)	3599.5 (2348.27)	3025.05 (1858.24)	0.3902
serine (mg) ***	2466.3 (2090.57)	2446.9 (1573.51)	1908.32 (1570.38)	0.5285
threonine (mg) ***	2189.7 (2364.33)	2057.57 (1210.06)	1711.9 (1111.92)	0.5014
tryptophan (mg) ***	699.62 (625.96)	670.24 (359.06)	542.65 (330.5)	0.5104
tyrosine (mg) ***	1796.03 (2101.56)	1758.21 (1458.56)	1405.41 (969.52)	0.4894

valine (mg) ***	2797.26 (2902.7)	2652.68 (1535.75)	2220.59 (1509.71)	0.5483
Lipids				
cholesterol (mg) ***	265.61 (242.32)	231.44 (197.22)	188.52 (197.6)	0.5786
SFA (g) *	32.15 (± 13.15)	29.95 (± 11.98)	26.75 (± 9.51)	0.203
Trans-FA (g) ***	0.22 (0.35)	0.28 (0.62)	0.31 (0.57)	0.7397
MUFA (g) ***	40.14 (15.53)	40.92 (21.17)	36.16 (15.62)	0.6583
Oleic acid (g) ***	30.69 (13.18)	29.89 (17.9)	31.41 (15.07)	0.9188
PUFA (g) ***	15.06 (9.34)	13.99 (8.43)	13.11 (7.28)	0.5029
Linolenic acid (g) ***	0.96 (0.63)	0.85 (0.48)	1.08 (0.69)	0.6863
Linoleic acid (g)***	11.31 (5.49)	10.07 (7.77)	10.93 (5.66)	0.5205
DHA (g) ***	0.03 (0.05)	0.02 (0.04)	0.03 (0.09)	0.3888
EPA (g) ***	0.01 (0.02)	0.01 (0.01)	0.01 (0.02)	0.6206
Total Dietary Fiber (g) ***	16.56 (11.75)	15.76 (9.24)	16.28 (14.31)	0.5338
crude fiber (g) ***	2.33 (2.74)	2.76 (2.62)	3.35 (3.9)	0.3724
insoluble fiber (g) ***	0.77 (1.74)	0.88 (1.5)	0.49 (1.49)	0.5367
soluble fiber (g) ***	0.14 (0.42)	0.16 (0.3)	0.08 (0.39)	0.6863
Minerals				
zinc (mg) ***	9.92 (6.17)	8.87 (5.47)	8.76 (3.13)	0.7386
copper (mg) ***	0.92 (0.47)	0.96 (0.43)	0.98 (0.58)	0.6293
chromium (mg) ***	0.04 (0.03)	0.02 (0.03)	0.04 (0.04)	0.08595
selenium (μg) *	111.27 (± 43.09)	111.71 (± 41.86)	107.01 (± 38.74)	0.889
manganese (mg) ***	2.06 (1.43)	1.96 (1.32)	1.68 (1.6)	0.5464
magnesium (mg) ***	251.14 (127.67)	231.54 (112.51)	245.04 (172.75)	0.7512
molybdenum (μg) ***	11.58 (10.82)	10.79 (22.87)	14.01 (32.02)	0.6124
iron (mg) ***	14.02 (6.44)	12.63 (8.12)	14.75 (5.57)	0.6666
iodine (μg) ***	4.5 (19.75)	5.22 (8.28)	5.83 (11.19)	0.857
calcium (mg) ***	863.59 (525.95)	768.77 (307.59)	751.72 (275.56)	0.5301
fluoride (μg) ***	169.05 (450.95)	438.26 (2405.9)	432.53 (1975.31)	0.093
phosphorus (mg) ***	1229.34 (665.25)	1075.22 (530.02)	1126.8 (454.76)	0.4303
potassium (mg) ***	2562.04 (1160.01)	2181.58 (941.84)	2468.09 (1063.26)	0.583
sodium (mg) ***	2318.32 (1089.37)	2100.77 (1167.93)	2043.72 (915.86)	0.3878
Vitamins				
Vitamin A (IU) ***	2405.9 (3533.26)	2934.74 (5660.69)	3636.52 (5472.72)	0.5298

Vitamin A (RAE) (µg) ***	294.3 (332.33)	369.74 (278)	308.65 (388.42)	0.6979
Vitamin A (RE) ***	403.26 (524.42)	484.87 (507.73)	679.05 (596.81)	0.5028
Vitamin C (mg) ***	49.19 (66.77)	44.17 (61.58)	60.67 (68.83)	0.6242
Vitamin D (µg) ***	2.18 (3.17)	2.79 (3.32)	2.19 (2.22)	0.5581
Vitamin D (IU) ***	59.26 (127.17)	108.25 (115.29)	80.37 (108.88)	0.3191
Vitamin E (mg) ***	1.09 (1.38)	0.31 (1.16)	0.82 (1.66)	0.3334
Vitamin E (IU) ***	0.62 (1.61)	0.46 (1.73)	1.1 (2.27)	0.4353
Vitamin K (µg) ***	56.11 (58.49)	70.85 (58.84)	85.11 (85.73)	0.2711
Cobalamin (Vitamin B12) (µg) ***	3.63 (2.83)	3.2 (2.05)	3.57 (2.66)	0.9548
biotin (µg) ***	10.8 (5.68)	9.45 (8.76)	10.65 (11.01)	0.7878
thiamin (mg) ***	1.66 (0.93)	1.64 (0.85)	1.35 (0.62)	0.2781
niacin (mg) ***	23.28 (17.76)	19.55 (13.49)	20.04 (11.48)	0.2629
pantothenic (mg) ***	3.83 (2.2)	3.59 (2.14)	3.56 (1.94)	0.7108
Pyridoxine (Vitamin B6) (mg) ***	1.7 (1.45)	1.43 (0.75)	1.62 (0.74)	0.1464
Alpha-Carotene (µg)***	150.4 (256.3)	159.45 (926.9)	118.12 (504.84)	0.503
Beta-Carotene (µg)***	701.98 (1793.47)	1122.85 (3264.89)	1001.74 (2892.92)	0.6092
Beta-Cryptoxanthin (µg) ***	10.71 (216.17)	81.2 (176.06)	36.72 (85.11)	0.6427
Lutein (+ Zeaxanthin) (µg) ***	780.5 (1143.24)	1008.71 (1157.6)	702.49 (1593.43)	0.76
lycopene (µg) ***	1099.39 (3429.34)	1857.86 (4087.54)	1711.41 (3327.42)	0.3552
Folate (DFE) (µg) ***	319.38 (170.7)	332.96 (215.89)	371.18 (267.01)	0.7513
Folate (Total) (µg) ***	255.79 (107.95)	283.09 (178.45)	311.63 (231.61)	0.8042
riboflavin (mg) ***	1.68 (1.15)	1.56 (1.09)	1.66 (0.72)	0.8107
Alpha-Tocopherol (mg) ***	8.94 (4.54)	7.92 (6.58)	7.96 (4.4)	0.717

Note: The normality assumption was checked using the Shapiro-Wilk test.; * parametric variable, *** non parametric variable.; Parametric quantitative variables are expressed as mean (± standard deviation (SD)) and non-parametric quantitative variables as median (interquartile range (IQR)); P value was obtained using Kruskal-Wallis with Dunn's post-hoc test or ANOVA with Tukey's post-hoc test for continuous non parametric and parametric variables, respectively; †Differences between low and high tertile, ‡Differences between medium and high tertile. ˄ Differences between low and medium tertile.

Table S3. Cont.

	Western pattern			
Nutrients	**Low**	**Medium**	**High**	**p-value**
protein (g) ***	81.39 (36.52)	96.87 (52.17)	97.44 (64.81)	0.4555
Total fat (g) ***	89.89 (38.61)	98.6 (44.85)	101.7 (39.58)	0.4902
carbohydrate (g) *	184.63 (± 63.27)	176.9 (± 55.14)	181.28 (± 63.97)	0.885
kilocalories (kcal) ***	1811.87 (532.82)	2044.97 (1055.28)	2077.82 (762)	0.4729
Total sugar (g) ***	62.63 (31.47)	59.1 (39.44)	51.02 (37.83)	0.4372
glucose (g) ***	10.8 (8.91)	7.32 (8.17)	6.77 (6.61)	0.1653
sucrose (g) ***	9.21 (10.32)	12.04 (14.9)	6.15 (14.33)	0.3743
maltose (g) ***	0.96 (1.03)	1.07 (0.71)	1.36 (1)	0.2107
fructose (g) ***	12.44 (13.01)	8.61 (14.46)	8.86 (7.79)	0.1244
galactose (g) ***	0.03 (0.21)	0.02 (0.08)	0.02 (0.09)	0.6883
lactose (g) ***	2.12 (5.54)	2.51 (6.49)	1.72 (6.65)	0.7513
Amino acids				
alanine (mg) ***	2026.68 (908.39)	2792.07 (2421.38)	2558.98 (2858.13)	0.1956
arginine (mg) ***	2212.67 (1018.99)	3139.43 (2880.97)	2913.86 (2972.27)	0.1234
aspartic acid (mg) ***	3701.22 (1588.1)	5416.59 (3831.69)	4752.96 (4482.39)	0.1405
cystine (mg) ***	581.74 (411.58)	779.86 (514.95)	732.07 (601.24)	0.1014
glutamic acid (mg) ***	8806.78 (5975.86)	11300.41 (8011.08)	11354.35 (8169)	0.07829
glycine (mg) ***	1756.36 (656.5)	2425.88 (1696.13)	2386.15 (2375.81)	0.1768
histidine (mg) ***	1236.88 (658.32)	1787.36 (1303.11)	1600.43 (1669.56)	0.1699
isoleucine (mg) ***	2002.97 (1180.13)	2687.32 (2092.19)	2440.25 (2269.61)	0.2
leucine (mg) ***	3376.28 (1785.96)	4619.62 (3451.67)	4367.89 (3999.92)	0.1708
lysine (mg) ***	2955.28 (1479.43)	4153.71 (3018.05)	3494.39 (4219.39)	0.2111
methionine (mg) ***	982.39 (552.10)	1343.35 (935.06)	1202.89 (1349.77)	0.2146

phenylalanine (mg) ***	1902.65 (1046.62)	2632.84 (1903.92)	2592.83 (2125.99)	0.1209
proline (mg) ***	2767.53 (2058.83)	3599.5 (1452.32)	3497.08 (2350.54)	0.1397
serine (mg) ***	1884.32 (1196.92)	2620.01 (1679.92)	2642.93 (2224.65)	0.08715
threonine (mg) ***	1711.9 (777.92)	2366.87 (1683.54)	2153.57 (1997.19)	0.1965
tryptophan (mg) ***	509.27 (317.35)	711.09 (535.33)	676.51 (551.84)	0.1425
tyrosine (mg) ***	1405.41 (766.11)	1950.9 (1461.35)	1959.33 (1945.98)	0.1946
valine (mg) ***	2220.59 (1273.79)	3162.98 (2172.47)	2812.21 (2604.39)	0.1889
Lipids				
cholesterol (mg) ***	202.35 (177.7)	249.98 (246.5)	227.41 (196.61)	0.3088
SFA (g) *	28.78 (± 10.17)	30.38 (± 13.45)	29.77 (± 11.85)	0.868
Trans-FA (g) ***	0.31 (0.58)	0.21 (0.3)	0.37 (0.62)	0.2512
MUFA (g) ***	35.79 (16.03)	40.92 (20.13)	40.97 (17.74)	0.373
Oleic acid (g) ***	29.35 (14.34)	30.12 (19.12)	31.6 (10.27)	0.626
PUFA (g) ***	13.45 (5.44)	15.58 (7.67)	14.06 (10.51)	0.383
Linolenic acid (g) ***	1.03 (0.75)	0.86 (0.52)	0.88 (0.76)	0.6267
Linoleic acid (g)***	10.93 (5.21)	11.56 (10.01)	11.08 (7.02)	0.1699
DHA (g) ***	0.02 (0.04)	0.04 (0.06)	0.02 (0.08)	0.2672
EPA (g) ***	0.01 (0.01)	0.02 (0.02)	0.01 (0.03)	0.8217
Total Dietary Fiber (g) ***	16.6 (10.42)	16.7 (13.74)	15.68 (9.02)	0.5673
crude fiber (g) ***	2.89 (2.74)	3.23 (2.95)	2.17 (2.96)	0.6959
insoluble fiber (g) ***	0.53 (0.84)	0.8 (1.53)	0.93 (1.73)	0.8177
soluble fiber (g) ***	0.12 (0.28)	0.16 (0.56)	0.04 (0.43)	0.5371
Minerals				
zinc (mg) ***	8.53 (3.55)	9.7 (7.28)	9.59 (4.52)	0.5955
copper (mg) ***	0.93 (0.52)	1.01 (0.49)	0.91 (0.47)	0.6231
chromium (mg) ***	0.03 (0.04)	0.03 (0.03)	0.04 (0.04)	0.7673
selenium (µg) *	106.05 (± 38.48)	111.82 (± 43.08)	112.55 (± 42.26)	0.796

manganese (mg) ***	1.86 (1.34)	2.05 (1.81)	1.79 (1.16)	0.9669
magnesium (mg) ***	243.35 (119.27)	234.41 (144.83)	247.89 (115.41)	0.7162
molybdenum (µg) ***	14.54 (38.6)	12.18 (23.85)	11.99 (10.37)	0.5234
iron (mg) ***	14.14 (5.67)	13.12 (7.49)	14.7 (7.79)	0.7626
iodine (µg) ***	8.54 (9.55) † ‡	7.2 (8.79) ‡	1.38 (4.47) †	**0.007605**
calcium (mg) ***	785.09 (466.31)	860.98 (433.43)	788.61 (290.27)	0.8876
fluoride (µg) ***	304.12 (1812.93)	457.07 (2444.13)	309.75 (1714.08)	0.471
phosphorus (mg) ***	1075.38 (539.15)	1268.39 (835.3)	1151.14 (436.12)	0.7482
potassium (mg) ***	2363.2 (1034.93)	2414.04 (1337.51)	2414.93 (1095.88)	0.6363
sodium (mg) ***	2045.51 (951.99)	2298.86 (1339.03)	2136.57 (896.47)	0.9447
Vitamins				
Vitamin A (IU) ***	6382.69 (8197.71) ለ†	2953.99 (4472.87) ለ	1949.61 (1201.88)†	**0.000329**
Vitamin A (RAE) (µg) ***	494.77 (451.33)ለ†	302.09 (280.74)ለ	248.02 (135.24)†	**0.000451**
Vitamin A (RE) ***	822.59 (772.66)ለ†	481.89 (443.18)ለ	370.68 (195.69)†	**0.000387**
Vitamin C (mg) ***	55.38 (59.73)	57.03 (63.43)	38.12 (71.01)	0.3145
Vitamin D (µg) ***	2.33 (2.4)	2.1 (3.2)	2.15 (3.01)	0.6516
Vitamin D (IU) ***	81.39 (110.16)	79.97 (122.35)	79.74 (115.13)	0.7426
Vitamin E (mg) ***	0.34 (1.2)	0.83 (1.62)	1.09 (1.52)	0.2825
Vitamin E (IU) ***	0.5 (1.79)	1.06 (2.01)	0.92 (1.58)	0.8507
Vitamin K (µg) ***	94.75 (97.54)ለ†	67.09 (31.43)ለ	56.11 (46.01)†	**0.03973**
Cobalamin (Vitamin B12) (µg) ***	3.73 (2.79)	3.42 (2.76)	3.59 (2.22)	0.9653
biotin (µg) ***	11.73 (9.46)	9.56 (9.63)	8.15 (9.96)	0.4747
thiamin (mg) ***	1.34 (0.58)	1.66 (1.06)	1.69 (0.86)	0.21
niacin (mg) ***	18.61 (10.32)	25.88 (15.22)	22.92 (16.98)	0.2185
pantothenic (mg) ***	3.11 (1.94)	3.86 (2.21)	3.8 (1.21)	0.4514

Pyridoxine (Vitamin B6) (mg) ***	1.46 (0.65)	1.77 (1.05)	1.55 (1.07)	0.4182
Alpha-Carotene (µg)***	484.94 (1161.97) †	158.03 (384.28)	96.55 (136.13) †	**0.02059**
Beta-Carotene (µg)***	3141.32 (3893.01)†	1122.85 (2539.55)	549.4 (639.54)†	**0.00293**
Beta-Cryptoxanthin (µg) ***	25.95 (79.17)	35.77 (175.04)	42.33 (220.41)	0.6338
Lutein (+ Zeaxanthin) (µg) ***	1227.21 (1736.94)†	844.63 (1157.6)‡	484.85 (633.56)†‡	**0.001501**
lycopene (µg) ***	1694.96 (3306.3)	1412.71 (3908.48)	1711.41 (4467.85)	0.8859
Folate (DFE) (µg) ***	371.18 (307.43)	309.2 (191.08)	295.5 (217.51)	0.4525
Folate (Total) (µg) ***	304.15 (214.83)	259.06 (154.65)	256.35 (129.61)	0.6209
riboflavin (mg) ***	1.66 (0.72)	1.57 (1.17)	1.61 (1.13)	0.8937
Alpha-Tocopherol (mg) ***	8.99 (2.86)	7.04 (8.37)	8.52 (4.18)	0.5452

Note: The normality assumption was checked using the Shapiro-Wilk test.; * parametric variable, *** non parametric variable.; Parametric quantitative variables are expressed as mean (± standard deviation (SD)) and non-parametric quantitative variables as median (interquartile range (IQR)); P value was obtained using Kruskal-Wallis with Dunn's post-hoc test or ANOVA with Tukey's post-hoc test for continuous non parametric and parametric variables, respectively; †Differences between low and high tertile, ‡Differences between medium and high tertile. ゟ Differences between low and medium tertile.

Table S3. Cont.

	High-Fat and Salt pattern			
**Nutrients**	**Low**	**Medium**	**High**	**p-value**
protein (g) ***	78.34 (43.66)	103.39 (49.3)	88.78 (49.97)	0.1311
Total fat (g) ***	79.86 (44.9)	99.51 (35.73)	98.61 (39.27)	0.06888
carbohydrate (g) *	172.94 (± 65.37)	179.67 (± 63.47)	190.29 (± 52.39)	0.543
kilocalories (kcal) ***	1638.67 (879.21)	2014.58 (668.75)	1974.61 (769.39)	0.112

Total sugar (g) ***	58.32 (39.98)	56.79 (37.44)	54.77 (32.37)	0.9644
glucose (g) ***	8.4 (11.71)	5.72 (8.52)	8.88 (6.93)	0.2593
sucrose (g) ***	9.66 (15.59)	8.35 (16.07)	8.59 (10.4)	0.7767
maltose (g) ***	1.11 (0.76)	0.88 (1.01)	1.21 (1.2)	0.2871
fructose (g) ***	10.5 (16.02)	7.66 (8.97)	10.3 (10.45)	0.2179
galactose (g) ***	0.02 (0.08)	0.01 (0.07)	0.04 (0.14)	0.1465
lactose (g) ***	2.51 (6.67)	1.09 (6.03)	2.64 (6.75)	0.9087
Amino acids				
alanine (mg) ***	2294.58 (1412.5)	2596.22 (2207.71)	2500.43 (2356.74)	0.5348
arginine (mg) ***	2443.7 (1840.23)	2685.31 (2617.04)	2879.44 (2427.72)	0.6016
aspartic acid (mg) ***	4374.41 (2637.64)	4664.19 (3553.33)	4647.87 (4103.66)	0.6378
cystine (mg) ***	613.78 (414.68)	700.39 (565.22)	737.48 (565.2)	0.4941
glutamic acid (mg) ***	9913.69 (5424.45)	11042.13 (8396.74)	11104.19 (7870.07)	0.4093
glycine (mg) ***	1914.91 (1307.92)	2064.51 (1766.44)	2222.83 (1860.1)	0.6303
histidine (mg) ***	1369.05 (1032.48)	1466.06 (1280.75)	1459.47 (1436.64)	0.5146
isoleucine (mg) ***	2079.34 (1327.21)	2242.27 (1968.31)	2299.87 (1915.1)	0.4861
leucine (mg) ***	3759 (2421.38)	3977.2 (3404.66)	3846.3 (3455.12)	0.4567
lysine (mg) ***	3237.88 (2525.33)	3662.4 (2987.31)	3459.23 (3530.06)	0.5415
methionine (mg) ***	1050.81 (767.06)	1198.88 (966.71)	1149.45 (1233.71)	0.6034
phenylalanine (mg) ***	2074.38 (1251.17)	2137.97 (2054.21)	2355.43 (1767.93)	0.3981
proline (mg) ***	3044.22 (1799.93)	3371.79 (2407.18)	3512.12 (2158.84)	0.3325
serine (mg) ***	2172.49 (1277.12)	2314.36 (1920.76)	2245.92 (1884.65)	0.3842
threonine (mg) ***	1942.93 (1214.99)	2037.13 (1608.3)	2022.06 (1737.06)	0.5161
tryptophan (mg) ***	553.9 (362.12)	595.67 (526.88)	656.78 (511.63)	0.3933
tyrosine (mg) ***	1581.27 (929.61)	1671.16 (1615.68)	1628.62 (1451.95)	0.5149
valine (mg) ***	2405.35 (1565.65)	2571.35 (2395.61)	2566.27 (2129.73)	0.4223
Lipids				

cholesterol (mg) ***	195.56 (136.88)	257.33 (268.7)	232.2 (186.71)	0.1147
SFA (g) *	25.5 (± 9.95)	32.32 (± 11.25)	30.88 (± 13.01)	0.0617
Trans-FA (g) ***	0.15 (0.24)ʎ†	0.35 (0.72)ʎ	0.46 (0.58)†	**0.002135**
MUFA (g) ***	32.27 (23.47)	41.66 (12.88)	39.55 (19.75)	0.1321
Oleic acid (g) ***	23.09 (15.1)	33.19 (12.48)	29.68 (11.19)	0.07164
PUFA (g) ***	14.55 (6.36)	15.02 (5.91)	14.01 (9.54)	0.6124
Linolenic acid (g) ***	0.86 (0.54)	0.9 (0.68)	0.99 (0.66)	0.6813
Linoleic acid (g)***	11.33 (6.19)	11.21 (6.02)	10.68 (5.75)	0.6986
DHA (g) ***	0.02 (0.04)	0.04 (0.07)	0.03 (0.14)	0.1784
EPA (g) ***	0.01 (0.02)	0.01 (0.02)	0.01 (0.07)	0.5602
Total Dietary Fiber (g) ***	17.55 (10.5)	14.1 (12.15)	15.75 (9.7)	0.9921
crude fiber (g) ***	3.23 (3.03)	2.25 (2.76)	2.97 (2.78)	0.7364
insoluble fiber (g) ***	0.5 (1.56)	0.58 (1.87)	0.77 (1.43)	0.6859
soluble fiber (g) ***	0.14 (0.28)	0.13 (0.42)	0.13 (0.45)	0.8722
Minerals				
zinc (mg) ***	8.58 (4.32)	10.69 (6.46)	9.14 (4.73)	0.1352
copper (mg) ***	0.96 (0.43)	0.93 (0.51)	0.98 (0.49)	0.8404
chromium (mg) ***	0.04 (0.04)	0.03 (0.03)	0.04 (0.03)	0.2449
selenium (µg) *	99.69 (± 38.48)	116.9 (± 41.67)	113 (± 41.63)	0.241
manganese (mg) ***	1.72 (1.34)	1.96 (1.07)	1.94 (1.64)	0.706
magnesium (mg) ***	242.9 (153.81)	239.11 (100.12)	254.17 (116.39)	0.7511
molybdenum (µg) ***	13.16 (30.52)	10.79 (29.76)	13.58 (16.72)	0.7835
iron (mg) ***	11.74 (7.15)	14.31 (6.4)	14.57 (8.11)	0.09348
iodine (µg) ***	6.85 (9.45)	5.03 (11.93)	4.93 (8.53)	0.9545
calcium (mg) ***	722.6 (409.65)	846.86 (487.97)	845.27 (348.86)	0.4507
fluoride (µg) ***	411.93 (5187.09)	273.08 (2553.03)	362.31 (545.97)	0.5384
phosphorus (mg) ***	920.33 (525.59)	1226.24 (552.54)	1150.27 (449.81)	0.2426

potassium (mg) ***	2415.86 (1075.97)	2345.19 (884.08)	2365.15 (1120.2)	0.7772
sodium (mg) ***	1748.31 (1051.08)	2339.78 (633.93)	2245.45 (1198.26)	0.09452
Vitamins				
Vitamin A (IU) ***	3867.95 (5139.67)	2704.66 (4766.89)	2405.13 (4404.59)	0.6296
Vitamin A (RAE) (µg) ***	382.96 (277.08)	319.52 (429.96)	267.62 (314.97)	0.4104
Vitamin A (RE) ***	617.44 (555.28)	522.97 (528.91)	411.67 (614.79)	0.6382
Vitamin C (mg) ***	86.58 (102.43)	47.61 (51.63)	44.11 (56.38)	0.2573
Vitamin D (µg) ***	2.03 (2.71)	2.13 (3.31)	2.93 (2.55)	0.7844
Vitamin D (IU) ***	62.72 (95.04)	74.59 (127.16)	109.33 (100.61)	0.3808
Vitamin E (mg) ***	0.7 (0.98)	0.7 (1.48)	0.82 (1.64)	0.3866
Vitamin E (IU) ***	0.54 (1.47)	0.48 (1.91)	1.02 (1.72)	0.2038
Vitamin K (µg) ***	73.41 (112.86)	66.99 (47.37)	68.45 (65.18)	0.3239
Cobalamin (Vitamin B12) (µg) ***	3.4 (1.75)	3.51 (3.66)	3.82 (2.34)	0.432
biotin (µg) ***	10.65 (8.35)	8.79 (9.69)	11.51 (10.24)	0.9215
thiamin (mg) ***	1.34 (0.76)	1.64 (0.79)	1.37 (0.86)	0.5773
niacin (mg) ***	19.55 (11.72)	21.46 (13.86)	22.73 (17.96)	0.6509
pantothenic (mg) ***	3.63 (2.18)	3.66 (2.33)	3.57 (2.37)	0.5595
Pyridoxine (Vitamin B6) (mg) ***	1.54 (1.01)	1.6 (0.86)	1.51 (0.97)	0.885
Alpha-Carotene (µg)***	190 (540.05)	174.07 (606.85)	90.52 (293.99)	0.287
Beta-Carotene (µg)***	1156.67 (3106.99)	955.93 (2719.11)	736.62 (2484.36)	0.4048
Beta-Cryptoxanthin (µg) ***	93.67 (153.41)	34.45 (134.13)	12.7 (73.96)	0.4264
Lutein (+ Zeaxanthin) (µg) ***	844.63 (1257.84)	721.19 (728.04)	708.04 (1535.33)	0.5777
lycopene (µg) ***	1300.81 (3433.08)	1849.15 (3160.32)	1857.98 (3780.65)	0.7103

Folate (DFE) (μg) ***	318.11 (233.85)	344.07 (271.78)	291.12 (190.17)	0.5795
Folate (Total) (μg) ***	261.98 (174.92)	288.11 (178.19)	268.68 (151.81)	0.7836
riboflavin (mg) ***	1.53 (0.72)	1.67 (1.08)	1.65 (1.04)	0.2017
Alpha-Tocopherol (mg) ***	6.92 (4.27)	8.42 (4.73)	8.94 (3.46)	0.4339

Note: The normality assumption was checked using the Shapiro-Wilk test.; * parametric variable, *** non parametric variable.; Parametric quantitative variables are expressed as mean (± standard deviation (SD)) and non-parametric quantitative variables as median (interquartile range (IQR)); P value was obtained using Kruskal-Wallis with Dunn's post-hoc test or ANOVA with Tukey's post-hoc test for continuous non parametric and parametric variables, respectively; †Differences between low and high tertile, ‡Differences between medium and high tertile. ᕱ Differences between low and medium tertile.

Table S3. Cont.

	Plant-based pattern			
**Nutrients**	**Low**	**Medium**	**High**	**p-value**
protein (g) ***	83.39 (46.53)	97.12 (59.32)	90.06 (41.98)	0.4334
Total fat (g) ***	90.83 (44.92)	98.38 (42.71)	98.6 (39.85)	0.475
carbohydrate (g) *	165.59 (± 48.56)	180.89 (± 56.9)	197.16 (± 70.92)	0.116
kilocalories (kcal) ***	1746.22 (798.9)	2078.76 (667.89)	1911.71 (717.21)	0.2124
Total sugar (g) ***	50.55 (33.73)	58.14 (34.43)	63.57 (31.09)	0.101
glucose (g) ***	6.52 (7.33)	6.77 (7.66)	10.37 (9.59)	0.1844
sucrose (g) ***	5.01 (12.48)†	7.46 (9.6) ‡	12.07 (14.71)† ‡	**0.01567**
maltose (g) ***	1.01 (0.71)	1.11 (1.07)	1.28 (1.46)	0.3777
fructose (g) ***	9.09 (9.1)	8.86 (14.91)	13.06 (13.84)	0.1069
galactose (g) ***	0.01 (0.1)	0.01 (0.07)	0.04 (0.18)	0.4291
lactose (g) ***	0.71 (5.82)	4.88 (6.76)	3.29 (5.99)	0.2927
Amino acids				
alanine (mg) ***	1993.34 (1994)	2647.96 (2289.32)	2294.58 (1714.32)	0.5555

arginine (mg) ***	2083.16 (2203.73)	3109.58 (2447.92)	2780.45 (2240.93)	0.3764
aspartic acid (mg) ***	3671.98 (3783.24)	4923.64 (3502.12)	4572.02 (3868.85)	0.5116
cystine (mg) ***	540.88 (436.72)	798.36 (576.3)	726.55 (478.64)	0.2469
glutamic acid (mg) ***	8806.78 (6865.09)	11929.38 (7730.93)	11319.84 (7364.84)	0.4077
glycine (mg) ***	1737.23 (1595.54)	2415.98 (1718.56)	2004.53 (1453.76)	0.3897
histidine (mg) ***	1242.83 (1364.7)	1600.83 (1353.42)	1369.05 (1176.26)	0.5603
isoleucine (mg) ***	2021.95 (1578.52)	2580.85 (1923.01)	2079.34 (1509.01)	0.4333
leucine (mg) ***	3500.22 (2659.5)	4542.32 (3355.75)	3759 (2725.61)	0.4739
lysine (mg) ***	2812.88 (3207.43)	3671.9 (2894.2)	3237.88 (2796.89)	0.5287
methionine (mg) ***	966.64 (891.74)	1242.18 (986.61)	1067.31 (848.35)	0.6096
phenylalanine (mg) ***	1964.99 (1453.52)	2662.47 (1776.84)	2359.57 (1584.91)	0.3256
proline (mg) ***	2982.18 (2222.47)	3477.52 (2315.6)	3603.29 (1688.19)	0.5077
serine (mg) ***	1905.64 (1541.95)	2713.87 (1986.59)	2293.4 (1688.82)	0.3193
threonine (mg) ***	1738.83 (1495.08)	2286.03 (1590.12)	1942.93 (1332.24)	0.5105
tryptophan (mg) ***	544.6 (404.4)	705.89 (516.48)	553.9 (426.8)	0.5013
tyrosine (mg) ***	1534.83 (1183.06)	1913.45 (1590.11)	1581.27 (1254.95)	0.4875
valine (mg) ***	2317.77 (1737.92)	2892.81 (2288.07)	2405.35 (1811.2)	0.4037
Lipids				
cholesterol (mg) ***	189.82 (165.18)	239.39 (172.8)	237.38 (202.07)	0.2595
SFA (g) *	29.07 (± 14.17)	30.91 (± 9.88)	29.08 (± 10.61)	0.802
Trans-FA (g) ***	0.31 (0.55)	0.3 (0.46)	0.18 (0.58)	0.6113
MUFA (g) ***	39.87 (20.05)	35.43 (17.43)	40.64 (16.88)	0.1958
Oleic acid (g) ***	28.39 (13.92)	26.5 (10.45)	33.93 (20.58)	0.2543
PUFA (g) ***	11.62 (7.1)	15.31 (7.88)	15.35 (6.39)	0.1266
Linolenic acid (g) ***	0.83 (0.59)	0.99 (0.64)	0.89 (0.71)	0.5862
Linoleic acid (g)***	8.68 (5.59)	11.53 (7.39)	11.33 (4.65)	0.109
DHA (g) ***	0.02 (0.04)	0.04 (0.08)	0.04 (0.06)	0.09831

EPA (g) ***	0.01 (0.01)	0.01 (0.02)	0.01 (0.02)	0.6998
Total Dietary Fiber (g) ***	12.9 (6.4)†	14.84 (9.23)‡	20.28 (12.83)† ‡	**0.00717**
crude fiber (g) ***	2.07 (2.49)	2.49 (2.51)	4.11 (2.87)	0.05848
insoluble fiber (g) ***	0.42 (1.2)	0.78 (1.32)	0.95 (1.82)	0.1065
soluble fiber (g) ***	0.11 (0.35)	0.14 (0.46)	0.16 (0.34)	0.4887
Minerals				
zinc (mg) ***	9.355.76	9.3 (4.58)	8.64 (6.13)	0.9957
copper (mg) ***	0.85 (0.27)†	0.96 (0.4)	1.11 (0.69)†	**0.01679**
chromium (mg) ***	0.04 (0.04)	0.03 (0.03)	0.04 (0.04)	0.216
selenium (µg) *	107.02 (± 43.34)	115.69 (± 45.13)	108.23 (± 34.87)	0.698
manganese (mg) ***	1.63 (0.75)	1.79 (1.22)	2.44 (2.89)	0.0939
magnesium (mg) ***	213.21 (77.08)	247.89 (123.64)	274.45 (127.32)	0.0613
molybdenum (µg) ***	19.87 (41.63)	7.35 (14.39)	11.88 (9.24)	0.4097
iron (mg) ***	11.26 (5.32)	14.21 (6.06)	15.53 (6.06)	0.07197
iodine (µg) ***	5.5 (8.79)	9.4 (15.95)	4.55 (7.88)	0.6181
calcium (mg) ***	709.08 (425.6)	816.66 (350.02)	860.98 (356.36)	0.6009
fluoride (µg) ***	242.55 (1464.25)	438.26 (2430.22)	403.15 (2276.24)	0.6267
phosphorus (mg) ***	1104.95 (606.46)	1151.14 (472.97)	1174.08 (564.34)	0.6214
potassium (mg) ***	2023.49 (1049.82)	2414.95 (919.19)	2584.54 (935.76)	0.1798
sodium (mg) ***	2120.27 (1020.58)	2474.68 (1290.7)	2050.04 (855.48)	0.3389
Vitamins				
Vitamin A (IU) ***	2183.04 (3393.2)	2783.41 (3630.28)	3867.95 (6611.13)	0.1154
Vitamin A (RAE) (µg) ***	271.2 (269.5)	317.52 (243.74)	469.19 (455.59)	0.1029
Vitamin A (RE) ***	422.11 (454.42)	464.63 (396.48)	700.24 (767.36)	0.2725
Vitamin C (mg) ***	50.37 (46.96)	42.73 (60.44)	91.77 (106.53)	0.183
Vitamin D (µg) ***	1.86 (2.36)	1.99 (3.66)	2.96 (2.23)	0.2145
Vitamin D (IU) ***	61.49 (79.62)	70.51 (104.13)	120.89 (99.12)	0.06926

Vitamin E (mg) ***	0.8 (1.14)	0.74 (1.31)	1 (1.79)	0.4037
Vitamin E (IU) ***	1.16 (1.72)	0.66 (1.58)	0.66 (1.99)	0.8972
Vitamin K (µg) ***	73.64 (62)	67.38 (57.7)	70.88 (81.3)	0.6202
Cobalamin (Vitamin B12) (µg) ***	3.18 (2.22)	3.59 (0.98)	3.71 (3.1)	0.7718
biotin (µg) ***	7.83 (7.53))†	10.78 (7.07)	12.83 (8.66))†	**0.0313**
thiamin (mg) ***	1.28 (0.72)	1.66 (0.86)	1.43 (0.73)	0.3257
niacin (mg) ***	18.23 (11.36)	22.98 (15.82)	21.94 (14.31)	0.2889
pantothenic (mg) ***	2.87 (1.61)†	3.71 (2.14)	4.18 (2.52)†	**0.01132**
Pyridoxine (Vitamin B6) (mg) ***	1.46 (1.09)	1.54 (0.77)	1.69 (0.98)	0.4405
Alpha-Carotene (µg)***	98.25 (233.78)	174.07 (509.91)	220.98 (641.25)	0.4201
Beta-Carotene (µg)***	559.9 (1563.2)	1035.76 (2725.7)	1082.14 (3385.19)	0.2847
Beta-Cryptoxanthin (µg) ***	13.51 (109.66)	25.95 (116.38)	61.71 (144.56)	0.5684
Lutein (+ Zeaxanthin) (µg) ***	721.19 (971.84)	1125.66 (1520.05)	676.2 (1215.03)	0.3676
lycopene (µg) ***	1437.21 (4034.55)	1375.66 (3359.76)	1857.86 (3567.55)	0.8029
Folate (DFE) (µg) ***	300.08 (203.41)	309.5 (182.54)	399.04 (276.24)	0.07691
Folate (Total) (µg) ***	239.45 (145.94)	257.83 (163.18)	322.33 (229.65)	0.05953
riboflavin (mg) ***	1.57 (0.66)	1.55 (0.72)	1.89 (0.86)	0.07028
Alpha-Tocopherol (mg) ***	7.4 (4.67)	7.06 (4.29)	9.39 (4.95)	0.1392

Note: The normality assumption was checked using the Shapiro-Wilk test.; * parametric variable, *** non parametric variable.; Parametric quantitative variables are expressed as mean (± standard deviation (SD)) and non-parametric quantitative variables as median (interquartile range (IQR)); P value was obtained using Kruskal-Wallis with Dunn's post-hoc test or ANOVA with Tukey's post-hoc test

for continuous non parametric and parametric variables, respectively; †Differences between low and high tertile, ‡Differences between medium and high tertile. ሉ Differences between low and medium tertile.

Table S3. Cont.

		Low-Fat Dairy and Poultry pattern		
**Nutrients**	**Low**	**Medium**	**High**	**p-value**
protein (g) ***	91.8 (39.23)	83.3 (49.48)	92.12 (58.58)	0.6765
Total fat (g) ***	91.46 (39.41)	91.46 (48.02)	102.66 (44.03)	0.703
carbohydrate (g) *	177.2 (± 58.71)	188.69 (± 62.68)	177.16 (± 60.89)	0.701
kilocalories (kcal) ***	1943.48 (639.79)	1887.51 (791.59)	1981.22 (993.7)	0.885
Total sugar (g) ***	53.22 (37)	52.33 (41.35)	62.76 (35.77)	0.403
glucose (g) ***	8.75 (7.59)	8.67 (7.34)	8.35 (8.4)	0.6399
sucrose (g) ***	13.92 (16.03)	7.08 (11.46)	9.13 (9.85)	0.5212
maltose (g) ***	1.11 (1.63)	1.16 (1.2)	1 (0.67)	0.6521
fructose (g) ***	10.44 (15.46)	9.6 (14.66)	10.97 (10.77)	0.9052
galactose (g) ***	0.02 (0.1)	0.06 (0.14)	0.01 (0.04)	0.8229
lactose (g) ***	3.1 (5.76)	0.48 (5.39)	4.83 (9.01)	0.1049
Amino acids				
alanine (mg) ***	2240.53 (1573.17)	2127.1 (1899.83)	2596.22 (2541.39)	0.7658
arginine (mg) ***	2610.62 (2021.92)	2612.07 (2029.28)	2936.48 (3030.26)	0.7951
aspartic acid (mg) ***	3763.37 (3559.25)	4499.7 (3699.54)	4942.99 (4210.13)	0.869
cystine (mg) ***	637.11 (503.41)	687.09 (400.8)	700.39 (620.85)	0.9936
glutamic acid (mg) ***	9953.36 (8229.91)	10034.83 (5811.46)	11176.01 (8685)	0.6345
glycine (mg) ***	2004.53 (1008.52)	1810.14 (1474.59)	2249.28 (2105.46)	0.8251
histidine (mg) ***	1262.16 (1125.63)	1331.09 (1280.59)	1540.5 (1647.12)	0.7372
isoleucine (mg) ***	2088.13 (1631.18)	2043.15 (1429.95)	2438.55 (2394.63)	0.7247
leucine (mg) ***	3618.52 (3108.99)	3679.54 (2420.87)	4328.54 (4315.58)	0.6442

lysine (mg) ***	3268.27 (2702.69)	3240.84 (2948.95)	3674.99 (3965.42)	0.6968
methionine (mg) ***	1074.34 (716.61)	1029.25 (840.83)	1198.88 (1286.24)	0.556
phenylalanine (mg) ***	2002.41 (1843.51)	2149.19 (1381.73)	2500.7 (2134.97)	0.7147
proline (mg) ***	3862.15 (2437.49)	3066.73 (1650.51)	3478.74 (2432.92)	0.3914
serine (mg) ***	2092.32 (1797.41)	2185.47 (1371.04)	2438.48 (1974.15)	0.717
threonine (mg) ***	1837.3 (1566.33)	1896.52 (1337.41)	2204.9 (1996.55)	0.7531
tryptophan (mg) ***	569.19 (435.33)	542.09 (370.08)	667.12 (577.22)	0.5152
tyrosine (mg) ***	1590.53 (1135.72)	1615.71 (1094.42)	1872.07 (1703.43)	0.4695
valine (mg) ***	2462.29 (1912.8)	2382.57 (1646.23)	2769.5 (2508.89)	0.6549
Lipids				
cholesterol (mg) ***	230.14 (179.32)	196.1 (175.98)	266.1 (219.9)	0.432
SFA (g) *	29.46 (± 10.49)	29.1 (± 13.71)	30.27 (± 11.07)	0.927
Trans-FA (g) ***	0.39 (0.58)	0.25 (0.46)	0.21 (0.68)	0.3783
MUFA (g) ***	39.73 (12.3)	35.42 (20.91)	42.35 (17.44)	0.2518
Oleic acid (g) ***	30.12 (10.72)	26.3 (13.41)	32.8 (17.46)	0.1163
PUFA (g) ***	14.83 (7.5)	12.93 (10.46)	15.21 (6.01)	0.8663
Linolenic acid (g) ***	0.94 (0.58)	1.02 (0.7)	0.84 (0.59)	0.8435
Linoleic acid (g)***	11.1 (7.05)	10.71 (6.59)	11.17 (6.52)	0.8679
DHA (g) ***	0.04 (0.08)	0.02 (0.04)	0.03 (0.05)	0.2132
EPA (g) ***	0.01 (0.04)	0.01 (0.02)	0.01 (0.01)	0.3633
Total Dietary Fiber (g) ***	15.92 (9.34)	16.61 (13.63)	15.34 (9.15)	0.6678
crude fiber (g) ***	2.51 (3.29)	3.23 (2.98)	2.53 (2.67)	0.4737
insoluble fiber (g) ***	0.42 (1.53)	0.55 (1.59)	0.97 (1.33)	0.2936
soluble fiber (g) ***	0.05 (0.31)	0.06 (0.4)	0.19 (0.42)	0.1521
Minerals				
zinc (mg) ***	9.33 (6.31)	9.05 (5.34)	9.09 (5.29)	0.2865
copper (mg) ***	1.02 (0.42)	0.91 (0.43)	0.94 (0.54)	0.5113

chromium (mg) ***	0.03 (0.04)	0.04 (0.04)	0.03 (0.03)	0.9497
selenium (μg) *	122.31 (± 41.89)	100.81 (± 38.39)	107.22 (± 40.62)	0.117
manganese (mg) ***	1.7 (1.48)	1.84 (1.17)	2 (1.53)	0.7559
magnesium (mg) ***	255.73 (126.46)	237.22 (116.64)	238.94 (132.33)	0.898
molybdenum (μg) ***	11.57 (9.16)	12.18 (47.88)	13.65 (27.45)	0.7567
iron (mg) ***	14.75 (6.75)	13.89 (7.21)	13.5 (6.03)	0.5084
iodine (μg) ***	7.2 (22.5)ለ	1.73 (5.46)ለ ‡	9.1 (8.57)‡	**0.006106**
calcium (mg) ***	776.69 (293.09)	707 (366.09)	890.05 (370.12)	0.05532
fluoride (μg) ***	276.94 (480.12)	425.77 (1824.01)	383.03 (4459.76)	0.771
phosphorus (mg) ***	1160.26 (546.73)	1056.78 (449.51)	1288.59 (599.27)	0.3065
potassium (mg) ***	2570.4 (987.52)	2360.32 (1224.09)	2416.9 (940.23)	0.9598
sodium (mg) ***	2389.26 (1434.03)	2033.13 (865.16)	2162.97 (1415.06)	0.3338
Vitamins				
Vitamin A (IU) ***	2934.74 (4613.83)	2681.09 (6203.26)	2617.63 (4022.31)	0.7727
Vitamin A (RAE) (μg) ***	340.86 (414.06)	262.16 (302.07)	368.82 (307.11)	0.3216
Vitamin A (RE) ***	451.08 (561.27)	512.98 (586.57)	489.92 (527.45)	0.971
Vitamin C (mg) ***	44.88 (65.77)	53.99 (60.01)	62.61 (91.42)	0.5134
Vitamin D (μg) ***	2.23 (1.85)	1.66 (2.94)	3.15 (3.2)	0.1125
Vitamin D (IU) ***	84 (78.08)	58.09 (102.37)	111.19 (124.49)	0.1296
Vitamin E (mg) ***	0.92 (1.45)	0.66 (1.63)	0.82 (1.14)	0.9228
Vitamin E (IU) ***	0.66 (1.79)	0.71 (1.96)	0.86 (1.4)	0.8476
Vitamin K (μg) ***	70.89 (51.77)	77.19 (113.73)	60.47 (50.95)	0.3737
Cobalamin (Vitamin B12) (μg) ***	3.66 (1.91)	2.25 (3.08)	3.65 (1.56)	0.1563
biotin (μg) ***	9.61 (8.92)	9.38 (9.9)	10.98 (7.95)	0.8822
thiamin (mg) ***	1.78 (0.78)	1.32 (0.78)	1.42 (0.81)	0.2771
niacin (mg) ***	24.55 (18.78)	17.29 (12.61)	21.43 (12.3)	0.159

pantothenic (mg) ***	3.63 (2.46)	3.51 (2.25)	3.73 (1.68)	0.7967
Pyridoxine (Vitamin B6) (mg) ***	1.47 (0.98)	1.57 (0.79)	1.6 (0.91)	0.9124
Alpha-Carotene (µg)***	220.98 (495.65)	151.37 (708.59)	84.54 (337.84)	0.5198
Beta-Carotene (µg)***	1155.99 (2674.2)	729.67 (2997.17)	686.15 (2282.08)	0.5218
Beta-Cryptoxanthin (µg) ***	27.87 (141.61)	12.52 (112.05)	60.43 (152.53)	0.3368
Lutein (+ Zeaxanthin) (µg) ***	1069.55 (905.36)	818.49 (1904.71)	571.21 (1071.86)	0.1882
lycopene (µg) ***	1286.5 (3891.61)	1377.54 (3344.56)	1857.98 (3939.82)	0.8602
Folate (DFE) (µg) ***	335.12 (251.05)	337.05 (127.39)	325.07 (221.62)	0.6739
Folate (Total) (µg) ***	287.62 (190.32)	274.96 (156.2)	253.24 (171.17)	0.5315
riboflavin (mg) ***	1.69 (0.98)	1.5 (0.73)	1.64 (0.88)	0.09782
Alpha-Tocopherol (mg) ***	8.77 (3.08)	7.98 (4.59)	8.39 (5.15)	0.8628

Note: The normality assumption was checked using the Shapiro-Wilk test.; * parametric variable, *** non parametric variable.; Parametric quantitative variables are expressed as mean (± standard deviation (SD)) and non-parametric quantitative variables as median (interquartile range (IQR)); P value was obtained using Kruskal-Wallis with Dunn's post-hoc test or ANOVA with Tukey's post-hoc test for continuous non parametric and parametric variables, respectively; †Differences between low and high tertile, ‡Differences between medium and high tertile. ᕱ Differences between low and medium tertile.

Table S4. The associations of the "High-sugar", "Prudent", "High-Fat and Salt" and "Plant-Based" patterns with the MRI parameters in the MAST4HEALTH obese and NAFLD patients.

	High-Sugar pattern					High-Sugar pattern	
	Low	Medium		High			
Variables		Beta (SE)	P value	Beta (SE)	P value	Beta (SE)	P value
Log- cT1 (ms)							
Model 1	Ref.	-0.009363 (0.022515)	0.678	0.016227 (0.022515)	0.473	0.007697 (0.009128)	0.401
Model 2	Ref.	-0.0095422 (0.0226940)	0.675	0.0160929 (0.0227151)	0.481	0.0080551 (0.0092216)	0.385
Model 3	Ref.	-0.0086118 (0.0221082)	0.698	0.0129563 (0.0221637)	0.56	0.0064641 (0.0090000)	0.4745
Model 4	Ref.	-5.309e-03 (2.361e-02)	0.8227	2.673e-02 (2.476e-02)	0.2839	1.061e-02 (1.003e-02)	0.2936
Model 5	Ref	-5.100e-03 (2.375e-02)	0.8306	2.893e-02 (2.545e-02)	0.2594	1.126e-02 (1.028e-02)	0.2769
Log-PDFF (%)							
Model 1	Ref.	0.05935 (0.17995)	0.742	0.21370 (0.18140)	0.242	0.11163 (0.07441)	0.137
Model 2	Ref.	0.058520 (0.176111)	0.7404	0.189930 (0.178059)	0.289	0.105188 (0.073345)	0.155
Model 3	Ref.	0.058877 (0.176845)	0.74	0.178793 (0.180153)	0.3237	0.100729 (0.074249)	0.17829
Model 4	Ref.	5.409e-02 (1.943e-01)	0.7815	2.406e-01 (2.066e-01)	0.248	1.108e-01 (8.486e-02)	0.1958
Model 5	Ref	5.144e-02 (1.955e-01)	0.7932	2.192e-01 (2.132e-01)	0.3074	1.022e-01 (8.748e-02)	0.2463
LIF							
Model 1	Ref.	-0.06037 (0.16042)	0.708	0.06206 (0.16042)	0.7	0.05167 (0.06479)	0.427
Model 2	Ref.	-0.061392 (0.162048)	0.706	0.060667 (0.162199)	0.709	0.052820 (0.065612)	0.423
Model 3	Ref.	-0.054358 (0.157235)	0.7304	0.036954 (0.157629)	0.8152	0.040937 (0.063815)	0.5229
Model 4	Ref.	-2.399e-02 (1.680e-01)	0.8868	1.109e-01 (1.762e-01)	0.5309	7.392e-02 (7.081e-02)	0.3
Model 5	Ref	-2.369e-02 (1.692e-01)	0.8890	1.141e-01 (1.813e-01)	0.5310	7.554e-02 (7.263e-02)	0.3018

	Prudent Pattern					Prudent Pattern	
	Low	Medium		High			
Variables		Beta (SE)	P value	Beta (SE)	P value	Beta (SE)	P value
Log- cT1 (ms)							
Model 1	Ref.	-0.02325 (0.02254)	0.305	-0.01587 (0.02218)	0.476	-0.012229 (0.008979)	0.177
Model 2	Ref.	'-0.0206597 (0.0232480)	0.377	-0.0140934 (0.0226033)	0.535	-0.0115590 (0.0092714)	0.216

Model 3	Ref.	-0.0298663 (0.0227096)	0.19188	-0.0244865 (0.0221650)	0.27229	-0.0121809 (0.0090016)	0.1794
Model 4	Ref.	-2.855e-02 (2.609e-02)	0.2775	-3.049e-02 (2.590e-02)	0.243	-1.744e-02 (1.021e-02)	0.0919
Model 5	Ref.	-2.835e-02 (2.621e-02)	0.2831	-2.812e-02 (2.631e-02)	0.2888	-1.659e-02 (1.059e-02)	0.1215
Log-PDFF (%)							
Model 1	Ref.	-0.2533 (0.1824)	0.168	-0.1707 (0.1811)	0.348	-0.06483 (0.07375)	0.382
Model 2	Ref.	-0.205675 (0.180812)	0.2583	-0.162368 (0.178287)	0.3649	-0.079181 (0.073286)	0.2828
Model 3	Ref.	-0.227887 (0.182969)	0.2162	-0.183651 (0.180312)	0.3112	-0.079797 (0.073518)	0.28064
Model 4	Ref.	-3.094e-01 (2.105e-01)	0.1458	-2.965e-01 (2.119e-01)	0.1658	-1.197e-01 (8.472e-02)	0.1618
Model 5	Ref.	-3.094e-01 (2.118e-01)	0.1485	-3.039e-01 (2.159e-01)	0.1636	-1.293e-01 (8.787e-02)	0.1456
LIF							
Model 1	Ref.	-0.13505 (0.16032)	0.402	-0.05614 (0.15772)	0.723	-0.08031 (0.06380)	0.211
Model 2	Ref.	-0.124743 (0.165544)	0.453	-0.049999 (0.160952)	0.757	-0.0798539 (0.0659564)	0.229
Model 3	Ref.	-0.191791 (0.161397)	0.23791	-0.125688 (0.157527)	0.42709	-0.084480 (0.063817)	0.18896
Model 4	Ref.	-1.224e-01 (1.835e-01)	0.507	-8.499e-02 (1.822e-01)	0.642	-7.607e-02 (7.211e-02)	0.2949
Model 5	Ref.	-1.216e-01 (1.847e-01)	0.5123	-7.563e-02 (1.854e-01)	0.6846	-7.397e-02 (7.480e-02)	0.3260

	**High-Fat and Salt pattern**					**High-Fat and Salt Pattern**	
	**Low**	**Medium**		**High**			
**Variables**		**Beta (SE)**	**P value**	**Beta (SE)**	**P value**	**Beta (SE)**	**P value**
Log- cT1 (ms)							
Model 1	Ref.	0.01811 (0.02258)	0.425	0.01626 (0.02222)	0.466	0.008804 (0.009045)	0.333
Model 2	Ref.	0.0176325 (0.0228271)	0.442	0.0140766 (0.0226289)	0.535	0.007833 (0.009257)	0.4
Model 3	Ref.	0.0160940 (0.0222091)	0.4706	0.0131197 (0.0220110)	0.5527	0.0063725 (0.0090296)	0.4822
Model 4	Ref.	1.465e-02 (2.411e-02)	0.5452	1.090e-02 (2.404e-02)	6.52E-01	7.338e-03 (9.989e-03)	0.4649
Model 5	Ref	1.587e-02 (2.440e-02)	0.5175	1.043e-02 (2.420e-02)	0.6678	7.065e-03 (1.008e-02)	0.4858
Log-PDFF (%)							
Model 1	Ref.	-0.06753 (0.18165)	0.711	0.14371 (0.17865)	0.423	0.05280 (0.07352)	0.474
Model 2	Ref.	-0.095125 (0.177561)	0.5935	0.133436 (0.175963)	0.4502	0.042520 (0.072888)	0.561
Model 3	Ref.	-0.100473 (0.178369)	0.5747	0.126750 (0.176882)	0.4755	0.039272 (0.073338)	0.5936
Model 4	Ref.	-1.046e-01 (1.960e-01)	0.595	1.214e-01 (1.968e-01)	0.539	0.0373 (8.308e-02)	0.6548

Model 5	Ref	-1.163e-01 (1.979e-01)	0.5585	1.295e-01 (1.982e-01)	0.5156	4.104e-02 (8.396e-02)	0.6264
LIF							
Model 1	Ref.	0.1964 (0.1594)	0.221	0.1721 (0.1568)	0.275	0.06297 (0.06417)	0.329
Model 2	Ref.	0.193896 (0.161391)	0.233	0.165007 (0.159990)	0.305	0.059625 (0.065787)	0.367
Model 3	Ref.	0.182495 (0.156484)	0.247	0.157915 (0.155088)	0.311	0.048755 (0.063958)	0.4479
Model 4	Ref.	1.811e-01 (1.691e-01)	0.288	1.024e-01 (1.687e-01)	0.5456	4.364e-02 (7.053e-02)	0.538
Model 5	Ref	1.864e-01 (1.713e-01)	0.2804	1.004e-01 (1.700e-01)	0.5566	4.296e-02 (7.125e-02)	0.5484

	**Plant-Based pattern**					**Plant-Based pattern**	
	**Low**	**Medium**		**High**			
**Variables**		**Beta (SE)**	**P value**	**Beta (SE)**	**P value**	**Beta (SE)**	**P value**
Log- cT1 (ms)							
Model 1	Ref.	-0.02379 (0.02219)	0.286	0.02309 (0.02163)	0.289	0.012699 (0.009007)	0.162
Model 2	Ref.	-0.0236373 (0.0231447)	0.31	0.025833 (0.0220403)	0.244	0.014794 (0.009246)	0.113
Model 3	Ref.	-0.0301260 (0.0226992)	0.1879	0.015908 (0.0218537)	0.4686	0.0096897 (0.0093605)	0.3034
Model 4	Ref.	-3.167e-02 (2.571e-02)	0.222	1.674e-02 (2.429e-02)	0.4931	7.841e-03 (1.050e-02)	0.4577
Model 5	Ref	-3.141e-02 (2.595e-02)	0.2302	1.642e-02 (2.457e-02)	0.5061	7.240e-03 (1.072e-02)	0.5016
Log-PDFF (%)							
Model 1	Ref.	-0.28777 (0.18137)	0.116	-0.02379 (0.17707)	0.893	0.01567 (0.07446)	0.834
Model 2	Ref.	-0.199071 (0.183026)	0.2796	0.003632 (0.175640)	0.9835	0.029830 (0.073881)	0.6873
Model 3	Ref.	-0.212570 (0.184686)	0.2528	-0.020352 (0.179768)	0.9101	0.018798 (0.076709)	0.807
Model 4:	Ref.	-1.394e-01 (2.120e-01)	0.5131	-1.250e-02 (2.057e-01)	0.9517	1.440e-02 (8.781e-02)	0.8702
Model 5	Ref	-1.401e-01 (2.132e-01)	0.5132	-1.699e-04 (2.085e-01)	0.9994	2.203e-02 (8.996e-02)	0.8072
LIF							
Model 1	Ref.	-0.1250 (0.1580)	0.431	0.1781 (0.1540)	0.251	0.09310 (0.06387)	0.148
Model 2	Ref.	-0.122907 (0.165354)	0.459	0.190982 (0.157464)	0.228	0.103658 (0.065770)	0.119
Model 3	Ref.	-0.171029 (0.161753)	0.2932	0.117376 (0.155728)	0.453	0.065216 (0.066372)	0.3285
Model 4	Ref.	-2.985e-01 (1.804e-01)	0.1024	3.907e-02 (1.705e-01)	0.8194	2.107e-02 (7.416e-02)	0.7771
Model 5	Ref	-3.004e-01 (1.821e-01)	0.1035	4.149e-02 (1.724e-01)	0.8106	1.958e-02 (7.574e-02)	0.7967

The cT1 (ms) and PDFF (%) were log-transformed due to the skewness of the distribution. Four adjustment sets were considered: Model 1: crude model; Model 2: adjusted for age + sex; Model 3: adjusted for Model 2 + BMI; Model 4: adjusted for Model 3 + PAL + smoking + center of the study + the other five dietary patterns; Model 5: adjusted for Model 4 + alcohol intake [yes/no]. A P value < 0.05 was considered significant in all tests. Ref: Reference (the low tertile of each dietary pattern was used as a reference group). Beta: beta coefficient. SE: standard error.
